# Verbal lie detection using Large Language Models

**DOI:** 10.1038/s41598-023-50214-0

**Published:** 2023-12-21

**Authors:** Riccardo Loconte, Roberto Russo, Pasquale Capuozzo, Pietro Pietrini, Giuseppe Sartori

**Affiliations:** 1 Molecular Mind Lab, IMT School for Advanced Studies Lucca, Piazza San Francesco 19, 55100 Lucca, LU Italy; 2 Department of Mathematics “Tullio Levi-Civita”, University of Padova, Padova, Italy; 3 Department of General Psychology, University of Padova, Padova, Italy

**Keywords:** Psychology, Human behaviour, Computer science

## Abstract

Human accuracy in detecting deception with intuitive judgments has been proven to not go above the chance level. Therefore, several automatized verbal lie detection techniques employing Machine Learning and Transformer models have been developed to reach higher levels of accuracy. This study is the first to explore the performance of a Large Language Model, FLAN-T5 (small and base sizes), in a lie-detection classification task in three English-language datasets encompassing personal opinions, autobiographical memories, and future intentions. After performing stylometric analysis to describe linguistic differences in the three datasets, we tested the small- and base-sized FLAN-T5 in three Scenarios using 10-fold cross-validation: one with train and test set coming from the same single dataset, one with train set coming from two datasets and the test set coming from the third remaining dataset, one with train and test set coming from all the three datasets. We reached state-of-the-art results in Scenarios 1 and 3, outperforming previous benchmarks. The results revealed also that model performance depended on model size, with larger models exhibiting higher performance. Furthermore, stylometric analysis was performed to carry out explainability analysis, finding that linguistic features associated with the Cognitive Load framework may influence the model’s predictions.

## Introduction

Lie detection involves the process of determining the veracity of a given communication. When producing deceptive narratives, liars employ verbal strategies to create false beliefs in the interacting partners and are thus involved in a specific and temporary psychological and emotional state^1^. For this reason, the Undeutsch hypothesis suggests that deceptive narratives differ in form and content from truthful narratives^2^. This topic has always been under constant investigation and development in the field of cognitive psychology, given its significant and promising applications in the forensic and legal setting^3^. Its potential pivotal role is in determining the honesty of witnesses and potential suspects during investigations and legal proceedings, impacting both the investigative information-gathering process and the final decision-making level^4^.

Decades of research have focused on identifying verbal cues for deception and developing effective methods to differentiate between truthful and deceptive narratives, with such verbal cues being, at best, subtle and typically resulting in both naive and expert individuals performing just above chance levels^5,6^. A potential explanation coming from social psychology for this unsatisfactory human performance is the intrinsic human inclination to the *truth bias*^7^*,* i.e., the cognitive heuristic of presumption of honesty, which makes people assume that an interaction partner is truthful unless they have reasons to believe otherwise^8,9^. However, it is worth mentioning that a more recent study challenged this solid result, finding that instructing participants to rely only on the best available cue, such as the detailedness of the story, enabled them to consistently discriminate lies from the truth with accuracy ranging from 59 to 79%^10^. This finding moves the debate on (1) the proper number of cues that judges should combine before providing their veracity judgment -with the suggestion that the use-the-best heuristic approach is the most straightforward and accurate- and thus on (2) the diagnosticity level of this cue.

More recently, the issue of verbal lie detection has also been tackled by employing computational techniques, such as stylometry. Stylometry refers to a set of methodologies and tools from computational linguistic and artificial intelligence that allow to conduct quantitative analysis of linguistic features within written texts to uncover distinctive patterns that can infer and characterize authorship or other stylistic attributes^11–13^. Albeit with some limitations, stylometry has been proven to be effective in the context of lie detection^14,15^. The main advantage is the possibility of coding and extracting verbal cues independently from human judgment, hence reducing the problem of inter-coder agreement, as researchers using the same technique for the same data will extract the same indices^15^.

Alongside this trend, several recent studies have explored computational analysis of language in different domains, such as fake news^16,17^, transcriptions of court cases^18–20^, evaluations of deceptive product reviews^21–23^, investigations into cyber-crimes^24^, analysis of autobiographical information^25^, and assessments of deceptive intentions regarding future events^26^. Taken together, most of those studies focused on the usage of Machine Learning and Deep Learning algorithms combined with Natural Language Processing (NLP) techniques to detect deception from verbal cues automatically (see Constâncio et al.^27^ for a systematic review of the computerized techniques employed in lie-detection studies).

More recently, a great step in advance has been made in the field of AI and NLP with the advent of Large Language Models (LLMs). LLMs are Transformer-based language models with hundreds of millions of parameters trained on a large collection of *corpora* (i.e., pre-training phase)^28^. Thanks to this pre-training phase, LLMs have proven to capture the intricate patterns and structures of language and develop a robust understanding of syntax, semantics, and pragmatics, being able to generate coherent text resembling human natural language. In addition, once pre-trained, these models can be fine-tuned on specific tasks using smaller task-specific datasets. Fine-tuning refers to the process of continuing the training of a pre-trained model on a new dataset, allowing it to adapt its previously learned knowledge to the nuances and specificities of the new data, thereby achieving state-of-the-art results^28^. Common tasks for LLMs fine-tuning include NLP tasks, such as language translation, text classification (e.g., sentiment analysis), question-answering, text summarization, and code generation. Therefore, LLMs excel at a wide range of NLP tasks, as opposed to models uniquely trained for one specific task^28^. However, to the best of our knowledge, despite the extreme flexibility of LLMs, the procedure of fine-tuning an LLM on small *corpora* for a lie-detection task has remained unexplored.

## Related works in the psychology field

Among previous psychological frameworks aimed at identifying reliable cues of verbal deception, the Distancing framework, the Cognitive Load (CL) theory, the Reality Monitoring (RM) framework, and the Verifiability Approach (VA) have been extensively studied, gaining empirical support for their efficacy not only from primary research but also from meta-analytic studies.

The Distancing framework of deception states that liars tend to distance themselves from their narratives as a mechanism to handle the negative emotions experienced while lying by using fewer self-references (e.g., "I," "me") and employing more other-references (e.g., "he," "they")^3,29^.

The CL framework states that liars consume more cognitive resources while fabricating their fake responses, checking their congruency with other fabricated information, and maintaining credibility and consistency in front of the examiner^30^, resulting in shorter, less elaborate, and less complex statements. A meta-analysis^31^ found that approaches based on CL theories produce higher accuracy rates in detecting deception than standard approaches.

The RM framework bases its assumptions on the memory characteristics literature hypothesizing that truthful recollections are based on experienced events, while deceptive recollections are based on imagined events^32^. Therefore, RM derives its predictions about truthful narratives from sensory, spatial, and temporal information and from emotions and feelings experienced during the event. On the contrary, predictions about deceptions are drawn from the number of cognitive operations (e.g., thoughts and reasonings)^33–35^. The total RM scores appear to be diagnostic (d = 0.55) in the detection accuracy of truthfulness^36,37^ (see also^38^ for an extensive review of verbal lie-detection methods). More recently, the RM framework was investigated through concreteness in language^39^. In this study, one underlying and partially supported assumption was the truthful concreteness hypothesis, which suggests that truthful statements usually consist of concrete, specific, and contextually relevant details. In contrast, deceptive or false statements often include more abstract and less specific information, being more associated with the RM criterion of cognitive operations.

The VA in verbal lie detection suggests that truthful statements are more likely to be verifiable than false or deceptive statements, as liars avoid mentioning details that could be verified with independent evidence to conceal their deception^40,41^. Verifiable details may be represented by activities involving or witnessed by identified individuals, documented through video or photographic evidence, or leaving digital or physical traces (e.g., phone calls or receipts)^40,41^.

Notably, these frameworks offer detectable linguistic cues that can be readily identified using NLP techniques and have been extensively studied in this sense.

Houch et al.^14^ conducted a meta-analysis of studies on computer-based lie detection, with most of the included studies relying on the Linguistic Inquiry and Word Count software (LIWC)^42,43^. LIWC is the gold standard tool for studying lexical diversity and text semantic content. Given a text, LIWC calculates the percentage of total words corresponding to more than 100 categories in the dictionary related to different psychosocial dimensions, which have been validated by human evaluators using rigorous procedures. Among Houch’s meta-analysis findings, LIWC metrics reflecting Distancing, CL, and RM frameworks of deception found support from the results and can detect verbal deception through computerized techniques.

Usually, for distancing metrics, researchers compute the number of self and other-references by summing the frequency of first-person pronouns in contrast with second and third-person pronouns^3,29^. When employing CL theory in texts, researchers usually employ and analyze statistics about the number of words and sentences, the readability, and the complexity of texts^12–14^. RM is often investigated with LIWC^26,44–46^. Schutte et al.^47^ provided evidence that human coding of perceptual and contextual details in discriminating lies from truths is not conclusively superior, thereby highlighting the potential advantages of automated techniques. Additionally, recent studies extracted verifiable details by using named-entity recognition (NER), proving to be an effective automatized procedure for the detection of deception in hotel reviews ^23^ as well as in participants’ intentions on their weekend plans^48^.

The promising results in applying NLP techniques for psychological research suggest the possibility of combining metrics from different psychological frameworks in a new theory-based stylometric analysis, offering the possibility to investigate verbal lie detection from multiple perspectives in one shot.

## Related works in the AI field

Previous works from the AI field have applied machine learning and deep learning models in a binary classification task for data-driven verbal deception detection.

Kleinberg and Verschuere^49^ developed a database of future intentions to investigate whether combining machine and human judgments may improve accuracy in predicting deception. While finding that human judgment impairs automated deception detection accuracy, the authors implemented two machine learning models (i.e., vanilla random forest) trained respectively on LIWC and Part-of-Speech features (e.g., frequency of names, adjectives, adverbs, verbs) reaching an accuracy of 69% (95% CI: 63–74%) and 64 (95% CI: 58%, 69%), respectively. On the same dataset, Ilias et al.^50^ evaluated six deep-learning models, including combinations of BERT (and RoBERTa), MultiHead Attention, co-attentions, and Transformers models. The best accuracy reached was 70.61% (± 2.58%) using a BERT with co-attention model. The authors also provided explainaibility analysis to understand how the models reached their decisions using a combination of LIME (a tool used to explain deep learning predictions in more straightforward and understandable terms by showing which specific words of the text influenced the outcome) and LIWC.

Capuozzo et al.^51^ developed a new cross-domain and cross-language dataset of opinions, asking English-speaking and Italian-speaking participants to provide truthful or deceptive opinions on five different topics. After encoding the texts with FastText word-embedding, they trained Transformers models in multiple scenarios using 10-fold cross-validation, with averaged accuracy ranging from 63% (± 8.7%) in the “within-topic” scenario to a high of 90.1% (± 0.16%) in the “author-based” scenario.

In contrast, Sap et al.^52^ developed a new dataset of narratives generated from memories and imagination and used an LLM (GPT-3) to compute a new metric called “sequentiality”. Sequentiality is a metric of narrative flow that compares the probability of a sentence with and without its preceding story context. While providing insights into the cognitive processes of storytelling with an innovative computational approach, the authors did not employ a fine-tuning procedure for an LLM to classify different narratives.

The findings in the AI domain indicate that as the model’s complexity increases, there is a heightened accuracy in predicting deception from texts. However, this increase in accuracy often comes at the expense of explainability for these predictions. LLMs are currently among the most cutting-edge models capable of handling vast amounts and complexities of linguistic data, and the lack of literature on fine-tuning LLMs for lie-detection tasks provides worthwhile reasons to investigate this area.

## Aims and hypotheses of the study

The main objectives and hypothesis of this study are outlined as follows:

- **Hypothesis 1a):** Fine-tuning an LLM can effectively classify the veracity of short narratives from raw texts, **1b)** outperforming classical machine learning and deep learning approaches in verbal lie detection.
- **Hypothesis 2):** Fine-tuning an LLM on deceptive narratives enables the model to also detect new types of deception;
- **Hypothesis 3):** Fine-tuning an LLM on a multiple-context dataset enables the model to obtain successful predictions on a multi-context test set;
- **Hypothesis 4):** Model performance depends on model size, with larger models showing higher accuracy;
- **Hypothesis 5a):** The linguistic style distinguishing truthful from deceptive statements varies across different contexts, **5b)** and can be a significant feature for model prediction.

To test Hypothesis 1a, we fine-tuned an open-source LLM, FLAN-T5, using three datasets: personal opinions (the Deceptive Opinions dataset^51^), autobiographical experiences (the Hippocorpus dataset^52^) and future intentions (the Intention dataset^49^). Given the extreme flexibility of LLMs, this approach is hypothesized to detect deception from raw texts above the chance level. To test the advantage of our approach compared to classical machine and deep learning models (Hypothesis 1b), we decided to compare the results with two benchmarks, further described in the Methods and Materials section.

With regards to Hypotheses 2 and 3, according to empirical evidence, classical machine learning models tend to experience a decline in performance when trained and tested on the aforementioned scenarios^53–55^. In contrast, LLMs have acquired a comprehensive understanding of language patterns during the pre-training phase. We posit that a fine-tuned LLM is capable of generalizing its learning across various contexts. Related to Hypothesis 4, we believe this generalization ability is further enhanced in larger models, as their size is associated with a more sophisticated representation of language.

Finally, to test Hypothesis 5, we introduced a new theory-based stylometric approach, named **D**e**CL**a**R**ati**VE** stylometry, to extract linguistic features related to the psychological frameworks of Distancing^29^, Cognitive Load^31^, Reality Monitoring^32^, and Verifiability Approach^40,41^, providing a pragmatic set of criteria to extract features from utterances. We will apply **D**e**CL**a**R**ati**VE** stylometry to compare truthful and deceptive statements in the three aforementioned datasets in order to explore potential differences in terms of linguistic style. Our hypothesis suggests that the linguistic style distinguishing truthful from deceptive statements may vary across the three datasets, as these types of statements originate from distinct contexts. We also applied the **D**e**CL**a**R**ati**VE** stylometry technique to provide explainability analysis of the top-performing model.

## Methods and materials

### Datasets

Three datasets were employed for this study: the Deceptive Opinions dataset^51^, from now on **Opinion Dataset**, the Hippocorpus dataset^52^, from now on **Memory Dataset**, and the **Intention dataset**^49^. For each dataset, participants were required to provide genuine or fabricated statements in three different domains: personal opinions on five different topics (Opinion dataset), autobiographical experiences (Memory dataset), and future intentions (Intention Dataset). Notably, the specific topic within each domain was counterbalanced among liars and truth-tellers. A more detailed description of each dataset is available in Supplementary Information as well as in the method section of each original article.

Table 1 displays an example of truthful and deceptive statements about opinions, memories, and intentions. Table 2 reports descriptive statistics for each dataset, both overall and when grouped by truthful and deceptive sets of statements. These statistics include the minimum, maximum, average, and standard deviation of word counts. Word counts were computed after text tokenization using spaCy, a Python library for text processing. Additionally, Table 2 provides Jaccard similarity index values between truthful and deceptive vocabulary sets. Jaccard's index was derived by calculating the intersection (common words) and union (total words) of these two sets^50,56^. The resulting index ranges from 0 to 1, with 0 indicating a completely different vocabulary between the two sets, and 1 indicating a completely identical vocabulary between the two sets. We reported the Jaccard similarity index to provide a measure of similarity or overlap between the word choices of truthful and deceptive statements within the respective datasets. Supplementary Information offers a detailed methodology for calculating the Jaccard similarity index.

**Table 1:** Truthful and deceptive example statements about opinions, memories, and intentions.

	Truthful	Deceptive
Opinion (Abortion)	While I am morally torn on the issue, I believe that ultimately it is a woman’s body and she should be able to do with it as she pleases. I belive people should not dehumanize the fetus tough, to make themselves feel better. The decision about laws regarding this issue should be left up to the states to decide. To combat this problem, birth control should be easily accessible	Abortion is the termination of a life and should not be al- lowed. If a fetus has made it to the point of being able to survive “on its own” outside its mother’s body, what right do we have to cut its life short. If the mother’s life is in danger, she already chose that she was willing to sacrifice her life to have a child when she consented to procreating
Memory (My boyfriend and I went to a concert together and had a great time. We met some of my friends there and really enjoyed ourselves watching the sunset.)	The day started perfectly, with a great drive up to Denver for the show. Me and my boyfriend didn’t hit any traffic on the way to Red Rocks, and the weather was beautiful. We met up with my friends at the show, near the top of the theater, and laid down a blanket. The opener came on, and we danced our butts off to the banjoes and mandolins that were playing on-stage. We were so happy to be there. That’s when the sunset started. It was so beautiful. The sky was a pastel pink and was beautiful to watch. That’s when Phil Lesh came on, and I just about died. It was the happiest moment of my life, seeing him after almost a decade of not seeing him. I was so happy to be there, with my friends and my love. There was nothing that could top that night. We drove home to a sky full of stars and stopped at an overlook to look up at them. I love this place I live. And I love live music. I was so happy	Concerts are my most favorite thing, and my boyfriend knew it. That’s why, for our anniversary, he got me tickets to see my favorite artist. Not only that, but the tickets were for an outdoor show, which I love much more than being in a crowded stadium. Since he knew I was such a big fan of music, he got tickets for himself, and even a couple of my friends. He is so incredibly nice and considerate to me and what I like to do. I will always remember this event and I will always cherish him. On the day of the concert, I got ready, and he picked me up and we went out to a restaurant beforehand. He is so incredibly romantic. He knew exactly where to take me without asking. We ate, laughed, and had a wonderful dinner date before the big event. We arrived at the concert and the music was so incredibly beautiful. I loved every minute of it. My friends, boyfriend, and I all sat down next to each other. As the music was slowly dying down, I found us all getting lost just staring at the stars. It was such an incredibly unforgettable and beautiful night
Intention (Going swimming with my daughter)	We go to a Waterbabies class every week, where my 16-month-old is learning to swim. We do lots of activities in the water, such as learning to blow bubbles, using floats to aid swimming, splashing and learning how to save themselves should they ever fall in. I find this activity important as I enjoy spending time with my daughter and swimming is an important life skill	I will be taking my 8-year-old daughter swimming this Saturday. We’ll be going early in the morning, as it’s generally a lot quieter at that time, and my daughter is always up early watching cartoons anyway (5 am!). I’m trying to teach her how to swim in the deep end before she starts her new school in September as they have swimming lessons there twice a week

In brackets, the topic assigned to the participant in the deceptive condition to fabricate the narrative.

**Table 2:** Summary statistics of the number of words for each dataset and truthful and deceptive set of statements.

Dataset (total number)	Min–Max number of words	Average number of words (SD)	Jaccard similarity Index (qualitative interpretation)
All opinions (2500)	6–338	59.05 (30.66)	0.35 (low similarity)
Truthful opinions (1250)	7–338	66.74 (31.95)	
Deceptive opinions (1250)	6–232	51.36 (27.24)	
All intentions (1640)	15–251	50.44 (30.11)	0.34 (low similarity)
Truthful intentions (783)	15–206	47.04 (28.36)	
Deceptive intentions (857)	15–251	53.55 (31.31)	
All memories (5506)	22–625	255.24 (92.36)	0.34 (low similarity)
Truthful memories (2770)	22–625	269.78 (94.14)	
Deceptive memories (2736)	22–609	240.51 (88.12)	

Jaccard Similarity Index and its qualitative interpretation in brackets refers to the similarity between truthful and deceptive vocabulary sets for each dataset.

### FLAN-T5

We adopted FLAN-T5, an LLM developed by Google researchers and freely available through HuggingFace Python’s library Transformers (https://huggingface.co/docs/transformers/model_doc/flan-t5). HugginFace is a company that provides free access to state-of-the-art LLMs through Python API. Among the available LLMs, we chose FLAN-T5 because of its valuable trade-off between computational load and goodness of the learned representation. FLAN-T5 is the improved version of MT-5, a text-to-text general model capable of solving many NLP tasks (e.g., sentiment analysis, question answering, and machine translation), which has been improved by pre-training^57^. The peculiarity of this model is that every task they were trained on is transformed into a text-to-text task. For example, while performing sentiment analysis, the output prediction is the string used in the training set to label the positive or negative sentiment of each phrase rather than a binary integer output (e.g., 0 = positive; 1 = negative). Hence, their power stands in both the generalized representation of natural language learned during the pre-training phase and the possibility of easily adapting the model to a downstream task with little fine-tuning without adjusting its architecture.

### DeCLaRatiVE stylometric analysis

This study employed stylometric analysis to achieve two primary objectives. First, we aimed to describe the linguistic features that distinguished the three datasets before initializing the fine-tuning process. Second, we conducted explainability analysis to gain insights into the role of linguistic style that differentiated truthful and deceptive statements in the model's classification process. For this purpose, a new framework that we referred to as **D**e**CL**a**R**ati**VE** stylometry was adopted, which involved the extraction of 26 linguistic features in conjunction with the psychological frameworks of **D**istancing^29^, **C**ognitive **L**oad^30,31^, **R**eality **M**onitoring^32,34^, and **VE**rifiability Approach^40,41^. A full list of the 26 linguistic features with a short description is shown in Table 3. This comprehensive approach enabled the analysis of verbal cues of deception from a multidimensional perspective.

**Table 3:** List and short description of the 26 linguistic features pertaining to the DeCLaRatiVE Stylometry technique.

Label	Description
num_sentences	Total number of sentences
num_words	Total number of words
num_syllables	Total number of syllables
avg_syllabes_per_word	Average number of syllables per word
fk_grade	Index of the grade level required to understand the text
fk_read	Index of the readability of the text
Analytic	LIWC summary statistic analyzing the style of the text in term of analytical thinking (0–100)
Authentic	LIWC summary statistic analyzing the style of the text in term of authenticity (0–100)
Tone	Standardized difference (0–100) of ‘tone_pos’—‘tone_neg’
tone_pos	Percentage of words related to a positive sentiment (LIWC dictionary)
tone_neg	Percentage of words related to a negative sentiment (LIWC dictionary)
Cognition	Percentage of words related to semantic domains of cognitive processes (LIWC dictionary)
memory	Percentage of words related to semantic domains of memory/forgetting (LIWC dictionary)
focuspast	Percentage of verbs and adverbs related to the past (LIWC dictionary)
focuspresent	Percentage of verbs and adverbs related to the present (LIWC dictionary)
focusfuture	Percentage of verbs and adverbs related to the future (LIWC dictionary)
Self-reference	Sum of LIWC categories ‘i’ + ‘we’
Other-reference	Sum of LIWC categories ‘shehe’ + ‘they’ + ‘you’
Perceptual details	Sum of LIWC categories ‘attention’ + ‘visual’ + ‘auditory’ + ‘feeling’
Contextual Embedding	Sum of LIWC categories ‘space’ + ‘motion’ + ‘time’
Reality Monitoring	Sum of Perceptual details + Contextual Embedding + Affect—Cognition
Concreteness score	Mean of concreteness score of words
People	Unique named-entities related to people: e.g., ‘Mary’, ‘Paul’, ‘Adam’
Temporal details	Unique named-entities related to time: e.g., ‘Monday’, ‘2:30 PM’, ‘Christmas’
Spatial details	Unique named-entities related to space: e.g., ‘airport’, ‘Tokyo’, ‘Central park’
Quantity details	Unique named-entities related to quantities: e.g., ‘20%’, ‘5 $’, ‘first’, ‘ten’, ‘100 m’

Features associated with the CL framework consisted of statistics about the length, readability, and complexity of the text^14,58–60^ and were extracted using the Python library TEXTSTAT. Features related to the Distancing and RM framework were computed using LIWC^42,43^, the gold standard software for analyzing word usage. Using the English dictionary, we scored each text along with all the categories present in LIWC-22. LIWC scoring was computed on tokenized text using the English dictionary. The selection of the LIWC categories related to the Distancing and RM framework was guided by previous research on computerized verbal lie-detection^29,49,50,52,56^ and a recent metanalysis^14^. RM was also investigated through linguistic concreteness of words^39^. To determine the average level of concreteness for each statement, we utilized the concreteness annotation dataset developed by Brysbaert et al.^61^. For the calculation of concreteness scores, a preprocessing pipeline was applied to textual data using the Python library SpaCy: text was converted to lowercase and tokenized; then stop words were removed, and the remaining content words were lemmatized. These content words were then cross-referenced with the annotated concreteness dataset to assign the respective concreteness value when a match was found. The concreteness score for each statement was then computed as the average of the concreteness scores for all the content words in that statement. For what concerns verifiable details, they were estimated by the frequency of unique named entities. Named entities were extracted with the NER technique using Python’s library SpaCy through the Transformer algorithm for English language (en_core_web_trf, https://spacy.io/models/en#en_core_web_trf).

Further details on how the 26 linguistic features were computed are provided in the Supplementary Information.

### Experimental set-up

**Figure 1:**
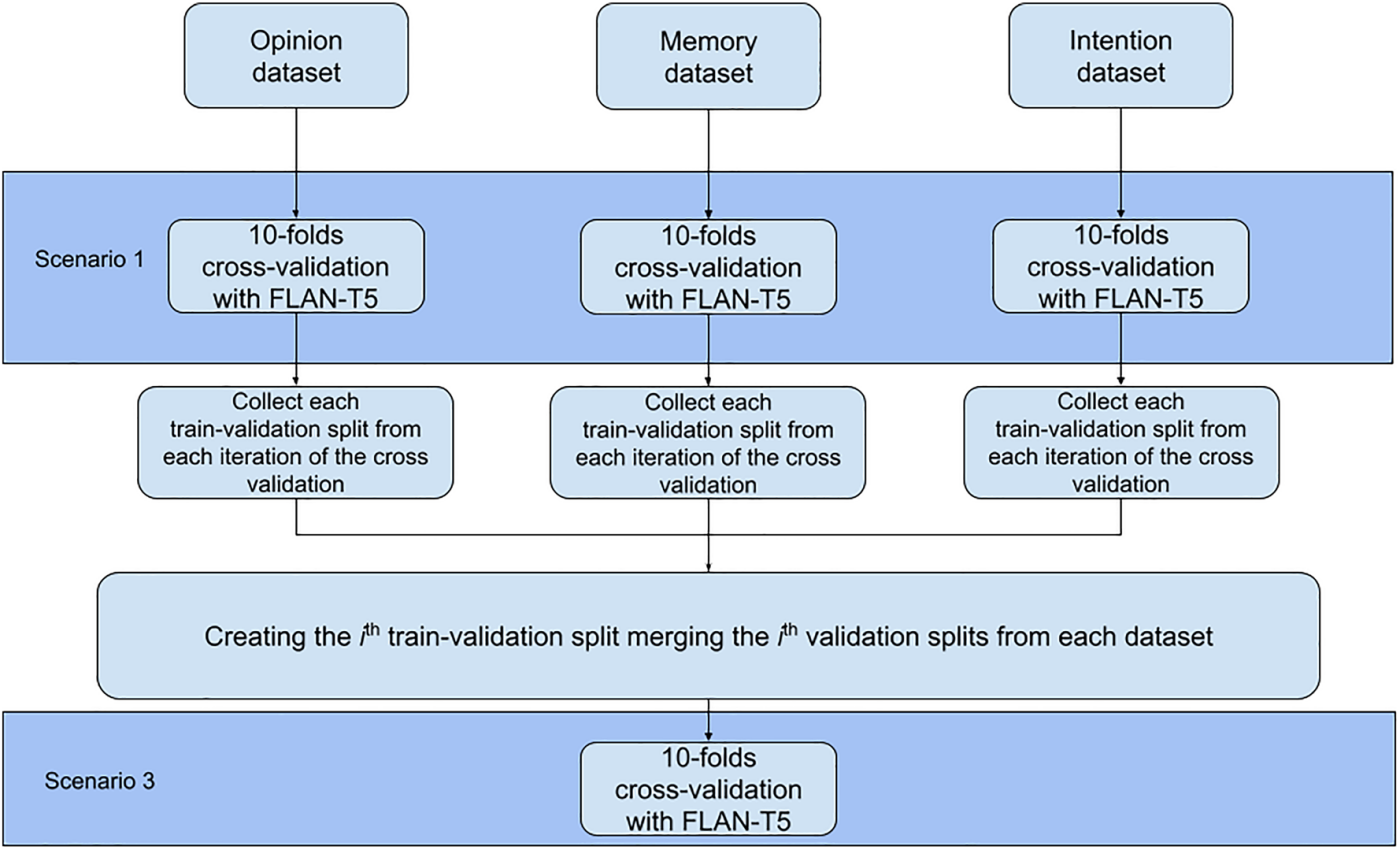
Visual illustration of the Scenarios 1 and 3.

**Figure 2:**
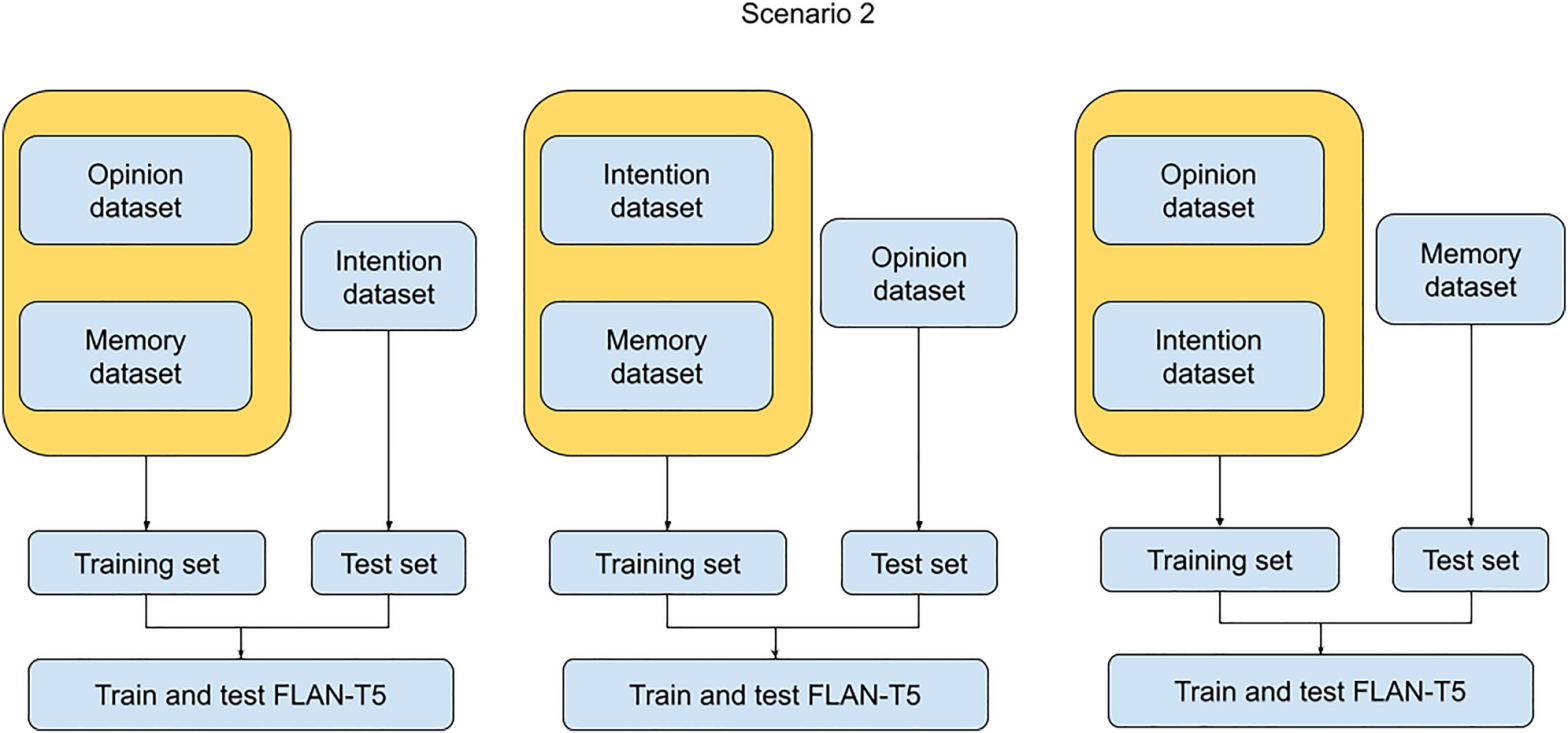
Visual illustration of the Scenario 2.

In this section, we describe the methodology that we applied in this work. As a first step, we wanted to perform a descriptive linguistic analysis of our datasets, trying to provide a response to Hypothesis 5a), i.e., whether the linguistic style distinguishing truthful from deceptive statements varies across different contexts. To achieve this result, we employed the **D**e**CL**a**R**ati**VE** stylometric analysis. As a second step, we proceeded to test the capacity of the FLAN-T5 model to be fine-tuned on a Lie Detection task. To do so, we provided three scenarios to verify the following hypothesis:

- **Hypothesis 1a):** Fine-tuning an LLM can effectively classify the veracity of short narratives from raw texts, **1b)** outperforming classical machine learning and deep learning approaches in verbal lie detection.
- **Hypothesis 2):** Fine-tuning an LLM on deceptive narratives enables the model to also detect new types of deception;
- **Hypothesis 3)**: Fine-tuning an LLM on a multiple-context dataset enables the model to obtain successful predictions on a multi-context test set;
- **Hypothesis 4):** Model performance depends on model size, with larger models showing higher accuracy;

We expected hypotheses 1a, 1b, 3, and 4 to be verified, while we did not have any a priori expectation for the second hypothesis. The scenarios are described below:

1. **Scenario 1:** The model was fine-tuned and tested on a single dataset. This procedure was repeated for each dataset with a different copy of the same model each time (i.e., the same parameters before the fine-tuning process) (Fig. 1). This Scenario assesses the model’s capacity to learn how to detect lies related to the same context and responds to Hypothesis 1a;
2. **Scenario 2:** The model was fine-tuned on two out of the three datasets and tested on the remaining unseen dataset. As for the previous Scenario, this procedure was iterated three times, employing separate instances of the same model, each time with a distinct combination of dataset pairings (Fig. 2). This Scenario assesses how the model performs on samples from a new context to which it has never been exposed during the training phase and provides a response for Hypothesis 2;
3. **Scenario 3:** We first aggregated the three train and test sets from Scenario 1. Then, we fine-tuned the model on the aggregated datasets and tested the model on the aggregated test sets (Fig. 1). This Scenario assesses the capacity of the model to learn and generalize from samples of truthful and deceptive narratives from multiple contexts and provides a response for Hypothesis 3.

In Scenarios 1 and 3, each experiment underwent a 10-fold cross-validation. N-fold cross-validation is a statistical method used to estimate the performance of a model by dividing the dataset into n partitions (n = 10 for this study). For each partition *i,* we created a training set composed of the remaining n−1 partitions using the *i* partition as a test set (i.e., 90% of the data belongs to the training set, and 10% of the remaining data belongs to the test set). For each iteration, performance metrics are computed on the test set, stored, and then averaged. This procedure ensures an unbiased performance estimation and allows a fair comparison between different models. For our study, we employed identical train-test splits within scenarios 1 and 3 and for both model sizes to guarantee a fair performance comparison. The average test accuracy from each fold and its corresponding standard deviation are presented as performance metrics. Conversely, in Scenario 2, each pairing combination underwent fine-tuning using the entire two paired datasets as a training set, while the model's performance was assessed using the complete unseen dataset as a test set.

Notably, the Opinion dataset was developed to have each participant's truthful and deceptive statements for a total of five opinions. Therefore, we treated each opinion as a separate sample. In order to avoid the model exhibiting inflated performance on the test set as a result of learning the participants’ linguistic style, we adopted the following precautionary measure. Specifically, we ensured an exclusive division of participants between the training and test sets, such that any individual who had their opinions assigned to the training set did not have their opinions assigned to the test set, and vice versa.

Together, Scenarios 2 and 3 provide evidence about the generalized capabilities of the fine-tuned FLAN-T5 model in a lie-detection task when tested on unseen data and on a multi-domain dataset. Furthermore, we tested whether model performance may depend on model sizes. Therefore, we first fine-tuned the *small*-sized version of FLAN-T5 in every scenario, and then we repeated the same experiments in every scenario with the *base*-sized version, providing a response for Hypothesis 4.

To test Hypothesis 1b, i.e., to test the advantage of our approach when compared to classical machine learning models, we decided to compare the results with two benchmarks:

1. A basic approach consisting of a bag-of-words (BoW) encoder plus a logistic regression classifier^62^ (following the experimental procedure of Scenario 1);
2. A literature baseline based on previous studies providing accuracy metrics on the same datasets using a machine learning or a deep learning approach^49–51^. For the Opinion dataset -characterized by opinions on five different topics per subject- we compared our results to the performance obtained in^51^ with respect to their “within-topic” experiments because our approach is equivalent to theirs, with the only difference that we addressed all the topics in one model.

As a final step, we conducted an explainability analysis to investigate the differences in linguistic style between the truthful and deceptive statements that were correctly classified and misclassified by the model. This procedure aimed to provide a response to Hypothesis 5b**,** i.e., whether the model takes into account the linguistic style of statements for its final predictions. To achieve this result, we employed the **D**e**CL**a**R**ati**VE** stylometric analysis.

**Figure 3:**
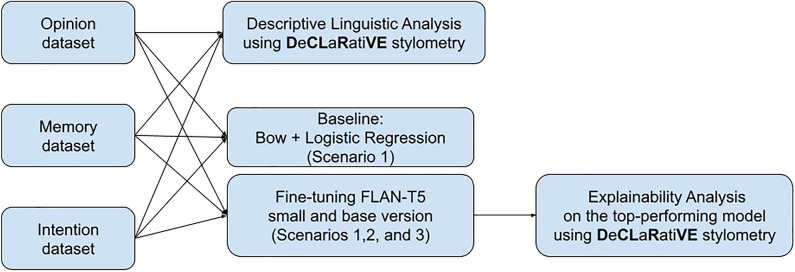
Visual illustration of the whole experimental set-up. The Opinion, Memory, and Intention dataset underwent Descriptive Linguistic Analysis using **D**e**CL**a**R**ati**VE** stylometry. A baseline model consisting of Bag of Words (BoW) and Logistic Regression (Scenario 1) was also established for the three datasets. Then, the FLAN-T5 model in small and base versions was fine-tuned across Scenarios 1, 2, and 3. Finally, an Explainability Analysis was conducted on the top-performing model using **D**e**CL**a**R**ati**VE** stylometry to interpret the results.

In Fig. 3, we provided a flow chart of the whole experimental set-up.

### Fine-tuning strategy

Fine-tuning of LLMs consists of adapting a pre-trained language model to a specific task by further training the model on task-specific data, thereby enhancing its ability to generate contextually relevant and coherent text in line with the desired task objectives^57^. We fine-tuned FLAN-T5 in its small and base size using the three datasets and following the experimental set-up described above. We approached the lie-detection task as a binary classification problem, given that the three datasets comprised raw texts associated with a binary label, specifically instances classified as truthful or deceptive.

To the best of our knowledge, no fine-tuning strategy is available in the literature for this novel downstream NLP task. Therefore, our strategy followed an adaptation of the Hugginface’s guidelines on fine-tuning an LLM for translation. Specifically, we chose the same optimization strategy used to pre-train the original model and the same loss function.

Notably, the classification task between deceptive and truthful statements has never been performed during the FLAN-T5 pre-training phase, nor is it included in any of the tasks the model has been pre-trained on. Therefore, we performed the same experiments, described in the Experimental set-up section, multiple times with different learning rate values (i.e., 1e−3, 1e−4, 1e−5), and we finally chose the configuration shown in Table 4, which yielded the best performance in terms of accuracy. All experiments and runs of the three scenarios were conducted on Google Colaboratory Pro + using their NVIDIA A100 Tensor Core GPU.

**Table 4:** FLAN-T5 hyperparameters configuration for the small- and base-sized version.

Model	Hyperparameter	Value
FLAN-T5 small	Learning rate	5e−4
	Weight decay coefficient	0.01
	Batch size	2
	Number of Epochs	3
FLAN-T5 base	Learning rate	5e−5
	Weight decay coefficient	0.01
	Batch size	2
	Number of Epochs	3

The initial learning rate for every scenario was 5e−4 for the small model and 5e−5 for the base model. This choice was motivated by preliminary experiment results, with the smaller model, but not the base model, generally performing better with higher learning rates. The weight decay coefficient was set to 0.01 in all models and Scenarios. The batch size was set to 2 for computational reasons, specifically to avoid running out of available memory, even though it is known that a larger batch size usually leads to better performance. Finally, the number of epochs was set to 3 after preliminary experiments showing the maximum test accuracy after the third epoch without overfitting.

## Statistical procedure for descriptive linguistic analysis

After applying the **D**e**CL**a**R**ati**VE** stylometry technique, we obtained a stylistic vector of 26 linguistic features for each text of the three datasets.

In order to assess the significance of the observed differences between the groups, a permutation t-test was employed^63^. This non-parametric method involves pooling all observations and then randomly redistributing them into two groups, preserving the original group sizes. The test statistic of interest (i.e., the difference in means) is then computed for these permuted groups. By repeating this process thousands of times (i.e., n = 10,000), we generated a test statistic distribution under the null hypothesis of no difference between the groups. The observed test statistic from the actual data was then compared to this distribution to compute a p-value, indicating the likelihood of observing such a difference if the null hypothesis was true. The advantage of using a permutation t-test is that no assumption about the distribution of data is needed. This analysis was conducted in Python using SciPy and Pingouin library.

For the Memory and Intention dataset, we computed a permutation t-test (n = 10,000) for independent samples for the 26 linguistic features to outline significant differences among the truthful and deceptive texts.

For the Opinion dataset, our analysis proceeded as follows. Firstly, we computed the **D**e**CL**a**R**ati**VE** stylometry technique for all the subjects’ opinions. This resulted in a 2500 (opinions) × 26 (linguistic features) matrix. Then, since each subject provided five opinions (half truthful and half deceptive), we averaged the stylistic vector separately for the truthful and deceptive sets of opinions. This procedure allowed us to obtain two different averaged stylistic vectors for the same subject, one for the truthful opinions and one for the deceptive opinions. Importantly, this averaging process enabled us to obtain results that are independent of the topic (e.g., abortion or cannabis legalization) and the stance taken by the subject (e.g., in favor or against that particular topic). Finally, we validated the statistical significance of these differences by conducting a paired sample permutation test (n = 10,000). Results for each dataset were corrected for multiple comparisons with Holm-Bonferroni correction.

The effect size was expressed by Common Language Effect Size (CLES) with a confidence interval of 95% (95% CI), which is a measure of effect size that is meant to be more intuitive in its understanding by providing the probability that a specific linguistic feature, in a picked-at-random truthful statement, will have a higher score than in a picked-at-random deceptive one^64^. The null value for the CLES is the chance level at 0.5 (in a probability range from 0 to 1) and indicates that, when sampled, one group will be greater than the other, with equal chance. Cohen’s *d* effect size with 95% CI was also computed to add interpretation.

## Statistical procedure for explainability analysis

To examine whether the linguistic style of the input statements exerted an influence on the resulting output of the model and to provide explanations for the wrong classification outputs, we applied a **D**e**CL**a**R**ati**VE** stylometric analysis of statements correctly classified and misclassified by the top-performing model identified in Scenario 3 (FLAN-T5 base).

To this aim, during each iteration from cross-validation, we paired the sentences belonging to the test set and their actual labels with the labels predicted by the model. After the cross-validation ended, for each of the ten folds and for each of the 26 linguistic features of the sentences that composed the test set for that fold, we performed a non-parametric permutation t-test for independent samples (n = 10,000) for the following comparison of interest:

- Truthful statements misclassified as deceptive (False Negatives), with deceptive statements misclassified as truthful (False Positives);
- Statements correctly classified as deceptive (True Negatives) vs. truthful statements misclassified as deceptive (False Negatives);
- Statements correctly classified as truthful (True Positives) vs. deceptive statements misclassified as truthful (False Positives).
- Truthful versus deceptive statements correctly classified by the model (True Positives vs. True Negatives).

To compute the effect size, we computed the average of the CLES and Cohen’s *d* effect size scores with their respective 95% CI obtained from each fold.

## Results

### Descriptive linguistic analysis

This section outlines the results of the descriptive linguistic analysis in terms of **D**e**CL**a**R**ati**VE** stylometric analysis to compare the three datasets on linguistic features.

**Figure 4:**
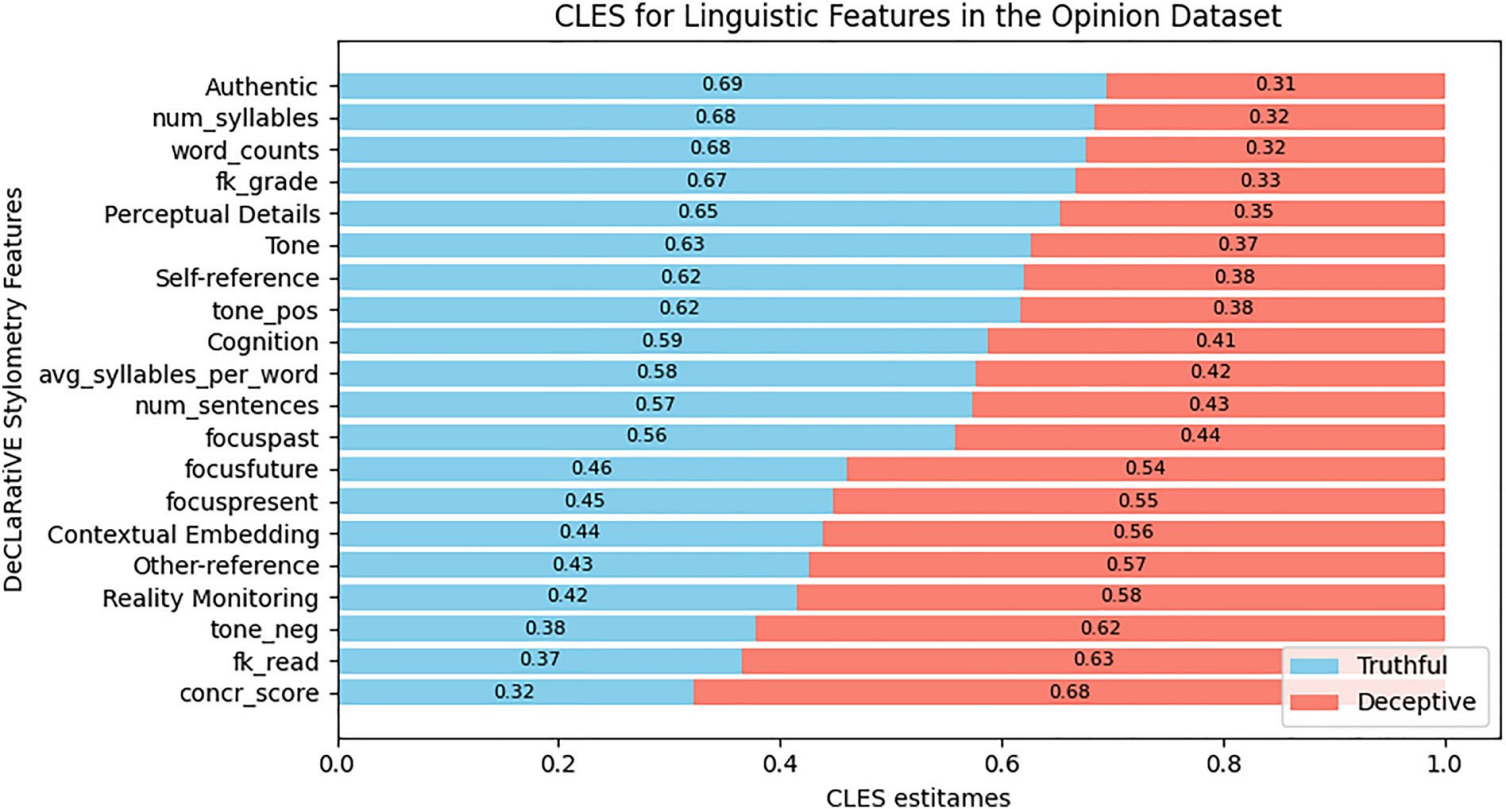
Horizontal stacked bar chart presenting the Common Language Effect Size (CLES) estimates for the significant linguistic features that survived post-hoc corrections in the Opinion dataset. The CLES estimates represent the probability (ranging from 0 to 1) of finding a specific linguistic feature in truthful opinions (sky blue) than in deceptive ones (salmon). The CLES for truthful opinions are sorted in descending order, while the CLES for deceptive opinions are sorted in ascending order.

**Figure 5:**
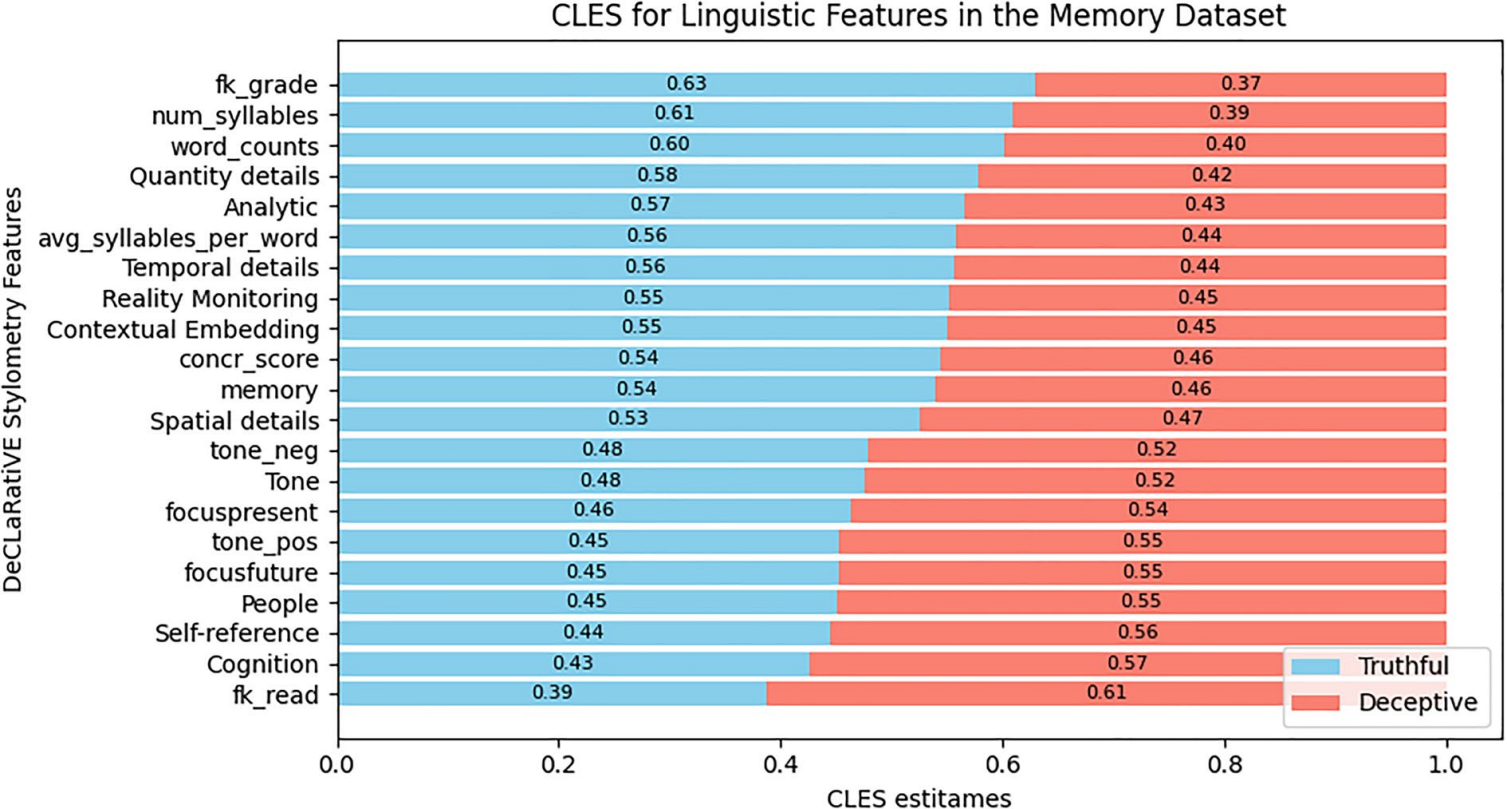
Horizontal stacked bar chart presenting the Common Language Effect Size (CLES) estimates for the significant linguistic features that survived post-hoc corrections in the Memory dataset. The CLES estimates represent the probability (ranging from 0 to 1) of finding a specific linguistic feature in truthful memories (sky blue) than in deceptive ones (salmon). The CLES for truthful memories are sorted in descending order, while the CLES for deceptive memories are sorted in ascending order.

**Figure 6:**
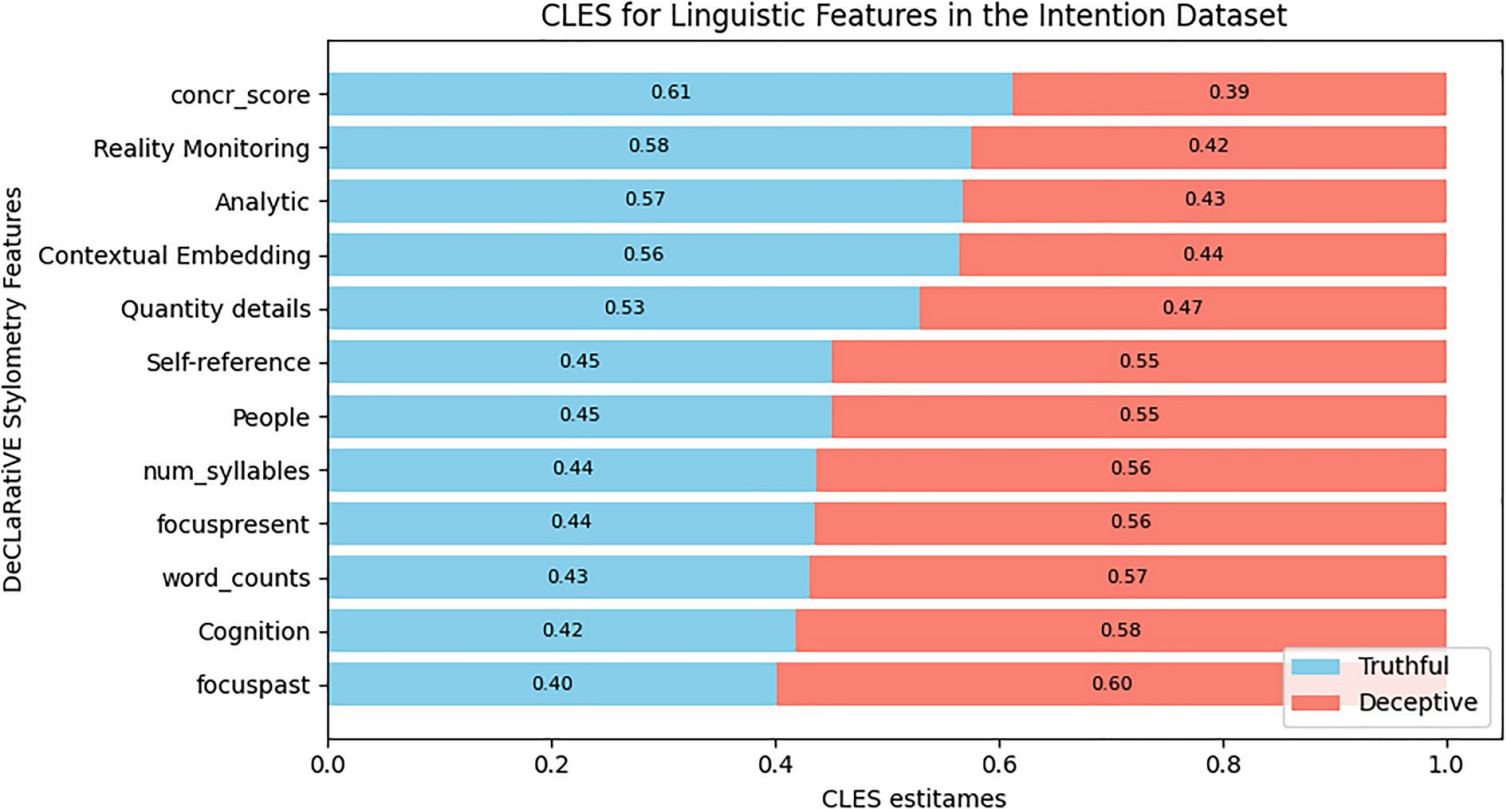
Horizontal stacked bar chart presenting the Common Language Effect Size (CLES) estimates for the significant linguistic features that survived post-hoc corrections in the Intention dataset. The CLES estimates represent the probability (ranging from 0 to 1) of finding a specific linguistic feature in truthful intentions (sky blue) than in deceptive ones (salmon). The CLES for truthful intentions are sorted in descending order, while the CLES for deceptive intentions are sorted in ascending order.

For the three datasets, Figs. 4, 5, and 6 show the differences in the number, the type, the magnitude of the CLES effect size, and the direction of the effect for the linguistic features that survived post-hoc corrections. To make an example of these differences, the concreteness score of words (‘concr_score’) presented the largest CLES within the Intention dataset towards the truthful statements (Fig. 6), while in the Opinion dataset, it showed the largest CLES towards the deceptive statements (Fig. 4). Overall, the Intentions dataset displayed fewer significant differences in linguistic features among truthful and deceptive statements than the Opinion and Memory datasets. In Table S5 (Supplementary Information), we reported, for all the linguistic features and the three datasets, all the statistics, the corrected p-values, the effect-size scores expressed by CLES and Cohen’s D with 95% CI, and the direction of the effect.

### Performance on the lie-detection classification task

This section presents the performance, in terms of averaged accuracy (and standard deviation) of the 10-folds, on the test sets after the last epoch of the small and base model in all the Scenarios.

#### Scenario 1

In Table 6 are depicted the test accuracies for the FLAN-T5 model, categorized by dataset and model size in Scenario 1. In each case, the base model, on average, outperformed the small model, with the Memory dataset showing the largest improvement of 4% and the Intention dataset showing just a 0.06% increase in average accuracy. These results indicate that the larger model size generally leads to improved performance across the three datasets, with higher accuracy observed in the base version.

#### Scenario 2

This scenario aimed to investigate our fine-tuned LLM’s generalization capability across different deception domains. As presented in Table 5, the test accuracy for the three experiments in this scenario significantly dropped to the chance level, showing that the model, in any case, was able to learn a general rule to detect lies coming from different contexts.

**Table 5:** Test accuracy of FLAN-5 models in scenario 2 (three combination of train sets).

Train set	Test set	Model size	Test accuracy
Opinion + Memory	Intention	FLAN-T5 small	55.37
		FLAN-T5 base	55.67
Opinion + Intention	Memory	FLAN-T5 small	55.37
		FLAN-T5 base	54.23
Memory + Intention	Opinion	FLAN-T5 small	53.12
		FLAN-T5 base	49.40

The performance comparison is among the small and base version of the FLAN-T5 model in the three combination of train set: opinion + memory, opinion + intention, memory + intention.

#### Scenario 3

In Scenario 3, we tested the accuracy of the FLAN-T5 small and base version on the aggregated Opinion, Memory, and Intention datasets. The small-sized FLAN-T5 achieved an average test accuracy of 75.45% (st. dev. ± 1.6), while the base-sized FLAN-T5 exhibited a higher average test accuracy of 79.31% (st. dev. ± 1.3). In other words, the base-sized model outperformed the small model by approximately four percentage points.

Results in Table 6 show the disaggregated performance on individual datasets between the small and base FLAN-T5 models in Scenario 3, with a comparison to their counterparts in Scenario 1. These comparisons show that FLAN-T5-small in Scenario 3 exhibited worse performance than in Scenario 1. Instead, in Scenario 3, the base model barely outperformed its counterparts of Scenario 1 on the Opinion and Intention datasets by less than 1% and slightly underperformed its counterpart of Scenario 1 on the Memory dataset.

**Table 6:** Test acccuracy of the FLAN-T5 models in Scenarios 1 and 3 for the three datasets.

Model	Opinion	Memory	Intention
Bag-of-words baseline	76.16 ± 2.9%	57.57 ± 7.66%	67.07 ± 3.18%
Literature baseline	65.16 ± 5.7%	–	69.00 [63; 74] % 69.86 ± 2.34% 70.61 ± 2.58%
FLAN-T5 small—Scenario 1	80.64 ± 2.03%	76.87 ± 2.06%	71.46 ± 3.65%
FLAN-T5 base—Scenario 1	82.60 ± 3.01%	**80.61 ± 1.41%**	71.52 ± 2.21%
FLAN-T5 small—Scenario 3	79 ± 2.11%	75.67 ± 1.90%	69.32 ± 3.75%
FLAN-T5 base—Scenario 3	**82.72** ± **2.39%**	79.87 ± 1.60%	**72.25** ± **2.86%**

Reported values are means ± standard deviation of the 10 folds. Best results per evaluation metric are in bold. The literature baseline for the Opinion dataset refers to the average accuracy and standard deviation from all within-topic accuracies from FastText Embedding + Transformer^51^. The literature baseline for the Intention dataset refers to the accuracy from Vanilla Random Forest using LIWC features (confidence interval in square brackets)^49^, the averaged accuracy and standard deviation from RoBERTa + Transformers + Co-Attention model and BERT + co-attention model^50^ respectively.

**Figure 7:**
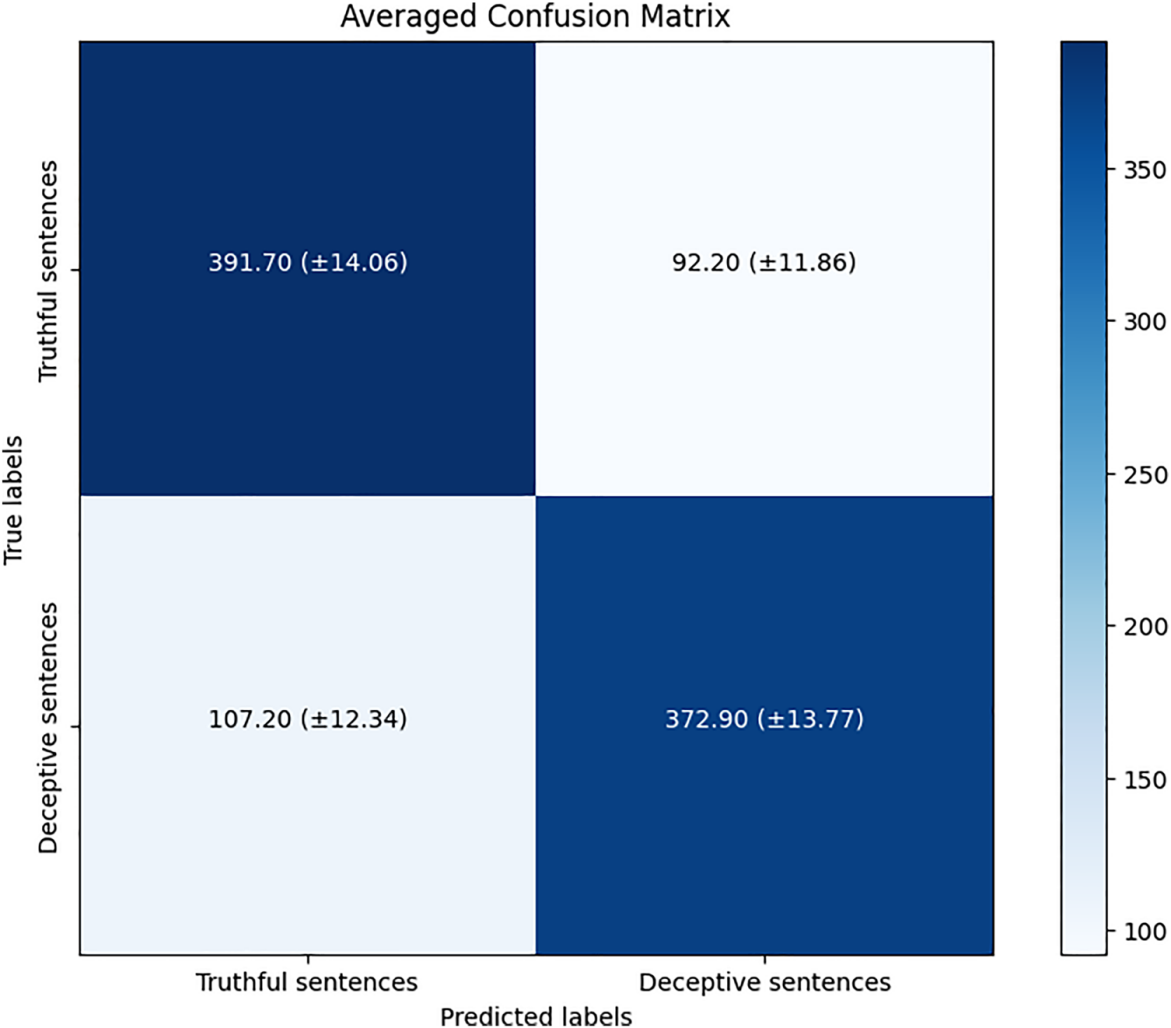
Averaged confusion matrix of the top-performing model identified as FLAN-T5 base in Scenario 3. In each square, the results obtained represent the average (and standard deviation) from the test set of each iteration of the 10-fold cross-validation.

We identified the top-performing model as the FLAN-T5 base in Scenario 3 because of its higher accuracy in the overall performance. The averaged confusion matrix of the 10 folds for this model is depicted in Fig. 7.

Notably, in any case, we were able to outperform both the bag of word + logistic regression classifier baseline and the performance achieved on the same datasets in previous studies^49–51^.

### Explainability analysis

This section aims to gain a deeper understanding of the top-performing model identified in Scenario 3 (FLAN-T5 base) through a **D**e**CL**a**R**ati**VE** stylometric analysis of statements correctly classified and misclassified by the model. The purpose of this analysis was to examine whether the linguistic style of the input statements exerted an influence on the resulting output of the model and to provide explanations for the wrong classification outputs. For this analysis, we compared:

- Truthful statements misclassified as deceptive (False Negatives), with deceptive statements misclassified as truthful (False Positives);
- Statements correctly classified as deceptive (True Negatives) vs. truthful statements misclassified as deceptive (False Negatives);
- Statements correctly classified as truthful (True Positives) vs. deceptive statements misclassified as truthful (False Positives).
- Truthful vs. deceptive statements correctly classified by the model (True Positives vs. True Negatives).

The statistically significant features reported survived post-hoc correction for multiple comparisons in each fold. Overall, for comparison a), b), and c), we observed no statistically significant differences (*p* < 0.05) in any linguistic features for most of the splits with the only exception of:

1. ‘fk_read’ in fold 1 (t = 5.30; *p* = 0.04, CLES = 0.63 [0.55, 0.71], d = 0.46 [0.18, 0.75]) and ‘Reality Monitoring’in fold 6 (t = 4.74; *p* = 0.047, CLES = 0.62 [0.54, 0.70], d = 0.46 [0.17, 0.75]) for the a) comparison;
2. ‘Reality Monitoring’in fold 6 (t = −3.39, *p* = 0.04, CLES = 0.40 [0.34, 0.46], d = −0.34 [−0.55, −0.13]) and ‘Reality Monitoring’ (t = −3.16 *p* = 0.04, CLES = 0.41 [0.34, 0.47], d = −0.34 [−0.56, −0.12]) and ‘Contextual Embedding’ (t = −2.11; *p* = 0.01, CLES = 0.39 [0.33, 0.45], d = −0.42 [−0.63, −0.2]) in fold 7 the b) comparison;
3. ‘num_syllables‘(t = 76.87, *p* = 0.01, CLES = 0.64 [0.57, 0.7], d = 0.46 [0.27, 0.7]) and ‘word_counts’(t = 59.63, *p* = 0.01, CLES = 0.64 [0.57, 0.71], d = 0.46 [0.21, 0.7]) in fold 9 for the c) comparison.

**Figure 8:**
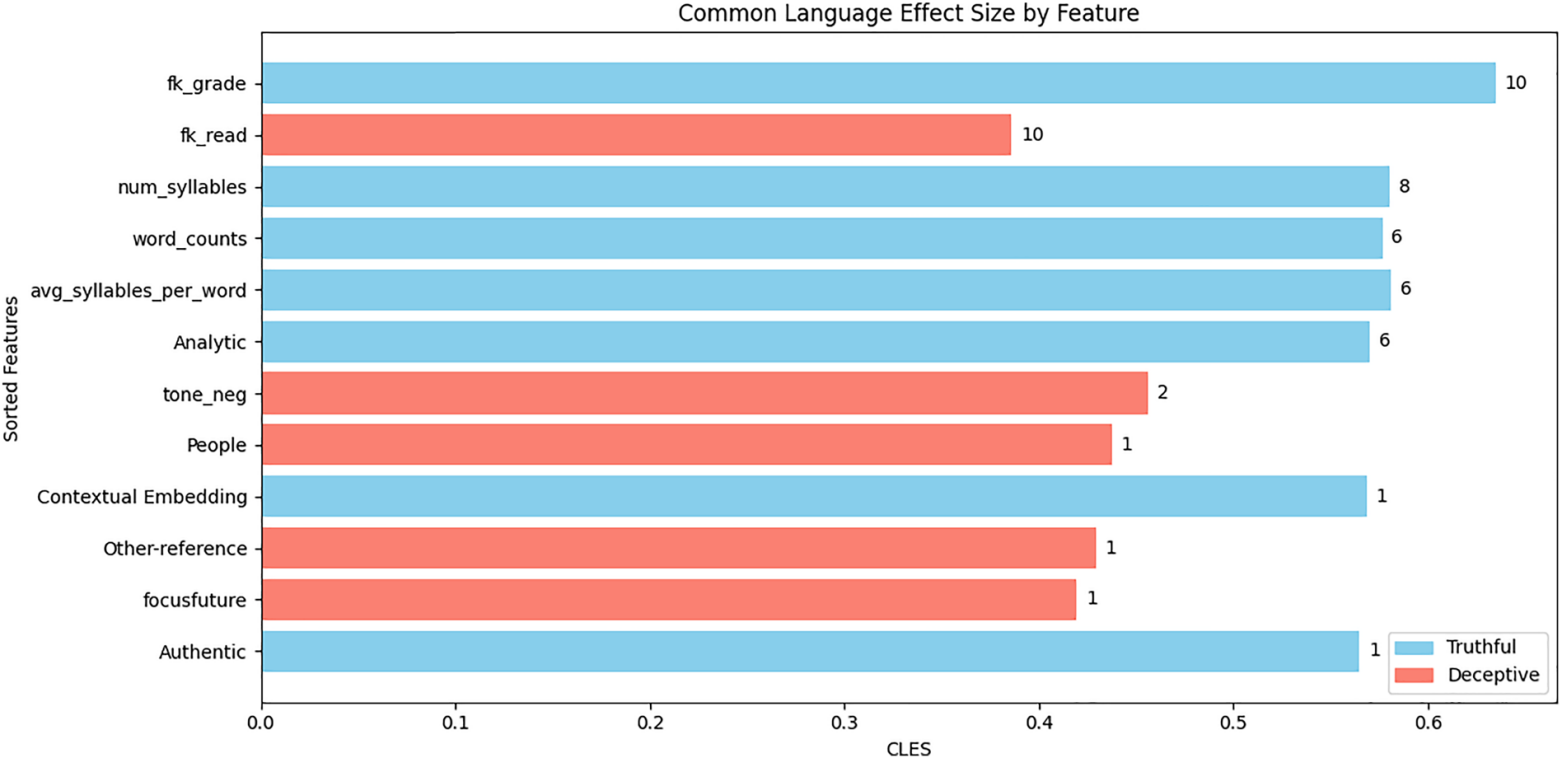
Linguistic features in Truthful and Deceptive statements that were accurately classified by FLAN-T5 base in Scenario 3. The bar plot shows the averaged Common Language Effect Size among the ten folds of linguistic features that survived post-hoc corrections. Linguistic features are sorted in descending order according to the number of times they were found to be significant among the 10 folds (displayed at the side of each bar). Linguistic features higher on average in truthful texts are shown in sky blue, while those higher on average in deceptive texts are shown in salmon.

Conversely, for the d) comparison, several significant features emerged in all the folds and survived corrections for multiple comparisons. Figure 8 depicts the CLES effect size scores of linguistic features, sorted according to the number of times they were found to be significant among the ten folds. The top six features in Fig. 8 represented a cluster of linguistic features related to the Cognitive Load framework.

## Discussion

In the present research, we investigated the efficacy of a Large Language Model, specifically FLAN-T5 in its small and base version, in learning and generalizing the intrinsic linguistic representation of deception across different contexts. To accomplish this, we employed three datasets encompassing genuine or fabricated statements regarding personal opinions, autobiographical experiences, and future intentions.

### Descriptive linguistic analysis

Descriptive linguistic analysis was performed to compare the three datasets on linguistic features by exploring the differences in the **D**e**CL**a**R**ati**VE** style, i.e., analyzing 26 linguistic features extracted from the psychological frameworks of **D**istancing, **C**ognitive **L**oad, **R**eality monitoring, and **VE**rifiability approach. This analysis aimed to test Hypothesis 5a, which postulates a variation in the linguistic style that differentiates truthful from deceptive statements across varying contexts (i.e., personal opinions vs. autobiographical memories vs. future intentions). The results from this analysis confirmed our hypothesis, showing that the linguistic features exhibiting statistically significant differences between truthful and deceptive statements indeed varied across datasets. This variation was observed in terms of the total number and type of features, the magnitude of the effect size (from very small to medium), and the direction of the effect. In the following paragraphs, the interpretation of the significant linguistic features of each dataset will be discussed.

### Opinions

After analyzing truthful and deceptive opinions using the **D**e**CL**a**R**ati**VE** stylometry, different linguistic features—related to the theoretical frameworks of CL, RM, and Distancing—were found to be significant.

In line with the CL framework, we observed that truthful opinions were characterized by greater complexity, verbosity, and more authenticity in linguistic style^14,31^.

For features related to the RM framework, truthful opinions were characterized by a lesser number of concrete words and a greater number of cognitive words, as also previously shown^55^; in contrast, deceptive opinions showed higher scores in the concreteness of words, contextual details, and reality monitoring. These differences may reflect on one side the reasoning processes that truth-tellers engage in evaluating the pros and cons of abstract and controversial concepts (e.g., abortion), while for deceivers, it may be indicative of difficulty in abstraction, resulting in faked opinions that sound more grounded in reality.

Finally, in line with previous literature on distancing framework^29,65^ and deceptive opinions^20,55^, deceivers utilized more other-related word classes (‘Other-reference’) and fewer self-related words (‘Self-reference’), confirming that individuals may tend to avoid personal involvement when expressing deceptive statements.

### Memories

Following the analysis of truthful and deceptive narratives of autobiographical memories through **D**e**CL**a**R**ati**VE** stylometry, various linguistic features associated with the theoretical frameworks of CL, RM, VA, and Distancing were found to be significant.

As for opinions, according to the CL framework, truthful narratives of autobiographical memories exhibited higher levels of complexity and verbosity and appeared to be more analytical in style^14,31^.

In accordance with the RM framework^32–37^, posing that truthful memory accounts tend to reflect the perceptual processes involved while experiencing the event while fabricated accounts are constructed through cognitive operations, we found genuine memories exhibiting higher scores in memory-related words and the number of words associated with spatial and temporal information (‘Contextual Embedding’), as well as an overall higher RM score. Conversely, we found deceptive memories showing higher scores in words related to cognitive processes (e.g., reasoning, insight, causation). Furthermore, in line with Kleinberg’s truthful concreteness hypothesis ^39^, truthful memories were overall characterized by words with higher scores of concreteness.

Along with the VA, truthful memories contained more verifiable details, as indicated by the greater number of named entities about times and locations^23,48^. Notably, we found this effect although participants lied in a low-stake scenario. However, deceptive memories were unexpectedly characterized by a higher number of self-references and named entities of ‘People’. This result is in contrast with previous literature on distancing framework^14,29^. One possible explanation of this significant but small effect is that liars may try to increase their credibility by fostering a sense of social connection.

### Intentions

Upon examining truthful and deceptive statements of future intentions through **D**e**CL**a**R**ati**VE** stylometry, several linguistic features were found to be significant. Our findings are consistent with previous research claiming that genuine intentions contain more ‘how-utterances’, i.e., indicators of careful planning and concrete descriptions of activities. In contrast, false intentions are characterized by ‘why-utterances’, i.e., explanations and reasons for why someone planned an activity or for doing something in a certain way^48^. Indeed, we found true intentions were more likely to provide concrete and distinct information about the intended action, grounding their statements in real-world experiences and providing temporal and spatial references. Additionally, true intentions were characterized by a more analytical style and a greater presence of numerical entities. In contrast, false intentions exhibited a higher number of cognitive words and expressions and were temporally oriented toward the present and past.

Furthermore, we found evidence in line with the claim that liars may over-prepare their statements^48^, as indicated by higher verbosity. Finally, in contrast with the distancing framework^14,29^, we found a significantly higher proportion of self-references and mentions of people in deceptive statements. However, the effect size for this finding was small. As for deceptive memories, one possible interpretation is that liars may attempt to appear more credible by creating a sense of social connection.

### Lie detection task

In order to test the capacity of the FLAN-T5 model to be fine-tuned on a Lie Detection task, we developed three scenarios.

In Scenario 1, we tested whether fine-tuning LLMs can effectively classify the veracity of short statements based on raw texts with performance highly above the chance level (Hypothesis 1a). To this aim, we fine-tuned FLAN-T5 in its small version to perform lie detection as a classification task. We repeated this procedure for the three datasets (i.e., opinions vs. memories vs. intentions). This fine-tuning process yielded promising results confirming our hypothesis, with an average accuracy of 80.64% (st. dev. ± 2.03%) for the Opinion dataset, 76.87% (st. dev. ± 2.06%) for the Memory dataset, and 71.46% (st. dev. ± 3.65%) for the Intention dataset.

In Scenario 2, we tested whether fine-tuning an LLM on deceptive narratives enables the model to detect new types of deception (Hypothesis 2). To verify this hypothesis, we fine-tuned FLAN-T5 (small version) on two datasets and tested on the third one (e.g., train: opinion + memory; test: intention). Our findings show that the model performed at chance level in all three combinations of this Scenario, suggesting that there are no universal rules the model can learn to distinguish truthful from deceptive statements, enabling a generalization of the task across different contexts. Indeed, as shown in the Descriptive Linguistic Analysis section, the three datasets differed significantly in terms of the content and the linguistic style by which truthful and deceptive narratives are delivered. Therefore, the model struggled to identify a specific pattern of linguistic deception and appeared to engage a domain-specific learning, tailoring its classification capabilities to that specific domain of deception.

In Scenario 3, we tested whether fine-tuning an LLM on a multiple-context dataset enables the model to obtain successful predictions on a multi-context test set (Hypothesis 3). At this aim, we fine-tuned and tested FLAN-T5 (small version) with the three aggregated datasets (i.e., opinion + memory + intention). The small-sized FLAN-T5 achieved an average accuracy of 75.45% (st. dev. ± 1.6). Additionally, the disaggregated performance on individual datasets compared to their counterpart in Scenario 1 exhibited solely a small decrease in accuracy (around 1%). These findings confirmed our hypothesis, providing evidence of LLMs’ ability to generalize when fine-tuned and texted on a multi-context dataset, in contrast to previous empirical evidence showing a decline in performance in machine learning models on the same scenarios^53–55^.

To test whether the model performance increases when employing larger models (Hypothesis 4), we repeated the same experiments in Scenarios 1, 2, and 3 with the base version of FLAN-T5.

In Scenario 1, we found that the base version of FLAN-T5 provided higher accuracy than the small version. In Scenario 3, the base version of the model achieved an average accuracy of 79.31% (st. dev. ± 1.3), outperforming the small model by approximately four percentage points. Additionally, this increase in the general accuracy did not compromise the performance on any individual dataset when compared to what achieved by the smaller model or by the FLAN-T5 base in Scenario 1. In contrast, the base version of FLAN-T5 in Scenario 2 still obtained performance around the chance level.

On one hand, the findings obtained from the base model in Scenarios 1 and 3 confirmed the hypothesis that the model size does influence the performance, likely because a bigger model is able to learn a better representation of linguistic patterns of genuine and deceptive narratives. Specifically, in Scenario 3, the FLAN-T5 base, with its larger size, possessed the capability to comprehend and integrate the features of the three distinct datasets altogether, thereby maintaining consistent performance across all individual datasets. In contrast, the smaller FLAN-T5 in Scenario 3 seemed to relinquish certain specialized abilities that are beneficial for specific datasets to classify deception across different contexts.

On the other hand, findings from Scenarios 2 and 3 (with small and base FLAN-T5) showed that LLMs, despite having acquired a comprehensive understanding of language patterns, still require exposure to prior examples to accurately classify deceptive texts within different domains.

Finally, to test whether our approach outperforms classical machine learning and deep learning approaches in verbal lie detection (Hypothesis 1b), we compared the results obtained from FLAN-T5 in its small and base versions with the performance of a simpler baseline of a logistic regressor based on BoW embedding^62^ and of Transformer models previously employed in the literature on the Opinion^51^ and Intention datasets^49,50^.

Specifically, when comparing the Memory dataset to the logistic regression baseline, there was a 32% increase in performance. This improvement might be attributed to the longer and more complex nature of the stories in the Memory dataset, which challenges the effectiveness of more straightforward methods like logistic regression based on BoW in a lie detection task. In contrast, LLMs already possess a robust language representation; thus, fine-tuning LLMs leverages this representation, tailoring their NLP proficiency specifically for a lie detection task, yielding higher accuracy.

The performance gained by fine-tuning LLMs was less pronounced for the Opinion and Intention datasets. For the Opinion dataset, this could be due to the relative ease of classification in these datasets, where simpler models can already achieve good performance, leaving a smaller margin for improvement. Nonetheless, the difference between our approach and the baselines is not negligible. In the Opinion dataset, we outperformed the literature baseline of a Transformer model trained from scratch by 17% accuracy and surpassed our logistic regression baseline by six percentage points. For the Intention dataset, our approach showed a 5-percentage point improvement over the logistic regression baseline and around 1–2% improvement over the best literature baseline. Notably, the best literature baseline for the Intention dataset (averaged accuracy: 70.61 ± 2.58%) used a similar approach to ours in terms of the type of model used, involving a Transformer-based model (BERT + Co-attention), which may explain the narrower performance gap.

Besides the differences in performance, the main advantage of our approach is its simplicity and flexibility compared to those used in previous studies^49–51^. Fine-tuning an LLM leverages an existing encoding of language that effortlessly handles any type of statement, unlike logistic regression based on BoW or training a new Transformer-based model from scratch. Taking all these aspects together, fine-tuning LLMs resulted in being more advantageous in terms of feasibility, flexibility, and performance accuracy.

### Explainability analysis

To improve the explainability of the performance collected, we investigated whether the linguistic style that characterizes truthful and deceptive narratives could have a role in the model’s final predictions (Hypothesis 5b). For this aim, we applied a **D**e**CL**a**R**ati**VE** stylometric analysis on statements that were correctly classified and misclassified by the top-performing model identified in Scenario 3 (i.e., FLAN-T5 base).

In the misclassified sample, truthful and deceptive statements did not differ significantly for any linguistic feature extracted with the **D**e**CL**a**R**ati**VE** stylometry technique. The only exception was fold 1, which showed significant differences in the text’s readability score, and fold 6, which showed significant differences in 'Reality Monitoring' scores. No significant differences were detected in each fold in linguistic features between deceptive statements that were correctly classified as deceptive (True Negatives) and truthful statements that were misclassified as deceptive (False Negatives), with the exception of ‘Reality Monitoring’ in folds 6 and 7 and ‘Contextual Embedding’ score in fold 7. Finally, truthful statements that were correctly classified as truthful (True Positives) and deceptive statements that were misclassified as truthful (False Positives) exhibited no significant differences, except for the number of syllables and number of words in the fold 9. We argue that the observation of significant differences in selected linguistic features across specific folds is more indicative that these findings may not be generalizable and are likely influenced by the particular fold under analysis. When taken together, most of the analyzed folds showed a substantial overlap in linguistic style. Consequently, the model might have exhibited poor classification performance for those statements because, while deceptive, they showed a linguistic style resembling truthful statements and vice-versa.

In contrast, correctly classified statements displayed several significant differences between truthful and deceptive statements. Notably, the top six linguistic features in Fig. 8 resulted in statistical significance in at least 6 out of 10 folds. The fact that we found a consistent pattern of linguistic features in correctly classified statements but not in misclassified statements provides evidence for our hypothesis, suggesting that the linguistic style of statements does have a role in the model’s final predictions. More in detail, the top-six linguistic features depicted in Fig. 8 represent a cluster of linguistic cues associated with the CL framework^31^, specifically low-level features related to the length, complexity, and analytical style of the texts that may have enabled the distinction between truthful and deceptive statements. The fact that linguistic cues of CL survived among the several features available -in a mixed dataset of utterances reflecting opinions, memories, and intentions- raises the question of whether CL cues may be more generalizable than other cues that are, in contrast, more specific to a particular type of deception.

## Conclusion, limitations, and further work

At the time of writing and to the best of our knowledge, this is the first study involving the use of an LLM for a lie-detection task.

LLMs are Transformer-based models trained on large corpora of text that have proven to generate coherent text in human natural language and have extreme flexibility in a wide range of NLP tasks^28^. In addition, these models can be further fine-tuned on specific tasks using smaller task-specific datasets, achieving state-of-the-art results^28^. In this study, we tested the ability of a fine-tuned LLM (FLAN-T5) on lie-detection tasks.

First, given the extreme flexibility of LLM, we tested whether fine-tuning a LLM is a valid procedure to detect deception from raw texts above chance level and outperform the classical machine and deep learning approaches. We found that fine-tuning FLAN-T5 on a single dataset is a valid procedure to obtain a state-of-the-art accuracy, as proved by the fact that this procedure outperformed the baseline model (BoW + logistic regression) and previous works that applied machine and deep learning techniques on the same datasets^49–51,62^.

Second, we wanted to investigate whether fine-tuning an LLM on deceptive narratives enables the model to also detect new types of deceptive narratives. Findings from Scenario 2 disconfirms this hypothesis, suggesting that the model requires previous examples of different deceptive narratives to provide adequate accuracy in this classification task.

Third, we investigated whether it is possible to successfully fine-tune an LLM on a multiple-context dataset. Results from Scenario 3 confirm that fine-tuned LLM may provide adequate accuracy in detecting deception from different contexts. We also found that fine-tuning on multiple datasets can increase the performance with respect to when fine-tuned on a single dataset.

Furthermore, we hypothesized that the model performance may depend on the model size, given that the larger the model, the better the model forms its inner representation of language. Results from Scenario 1 and 3 confirmed that the base-sized model of FLAN-T5 provides higher accuracy than the small-sized version.

Finally, with our experiments, we introduced the **D**e**CL**a**R**ati**VE** stylometry technique, a new theory-based stylometric approach to investigate deception in texts from four psychological frameworks (Distancing, Cognitive Load, Reality Monitoring, and Verifiability approach). We employed the **D**e**CL**a**R**ati**VE** stylometry technique to compare the three datasets on linguistic features and we found that fabricated statements from different contexts exhibit different linguistic cues of deception. We also employed the **D**e**CL**a**R**ati**VE** stylometry technique to conduct an explainability analysis and investigate whether the linguistic style by which truthful or deceptive narratives are delivered is a feature that the model takes into account for its final prediction. At this aim, we compared correctly classified and misclassified statements by the top-performing model (FLAN-T5 base in Scenario 3), finding that correctly classified statements share linguistic features related to the cognitive load theory. In contrast, truthful and deceptive misclassified statements do not present significant differences in linguistic style.

Given the results achieved, we highlight the importance of a diversified dataset to achieve a generalized good performance. We also considered crucial the balance between the diversity of the dataset and the size of the LLM, suggesting that the more diverse the dataset is, the bigger the model required to achieve higher-level accuracy. The main advantage of our approach consists of its applicability to raw text without the need for extensive training or handcrafted features.

Despite the demonstrated success of our model, three significant limitations impact the ecological validity of our findings and their practical application in real-life scenarios.

The first notable limitation pertains to the narrow focus of our study, which concentrated solely on lie detection within three specific contexts: personal opinions, autobiographical memories, and future intentions. This restricted scope limits the possibility of accurately classify deceptive texts within different domains. A second limitation is that we exclusively considered datasets developed in experimental set-ups designed to collect genuine and completely fabricated narratives. However, individuals frequently employ embedded lies in real-life scenarios, in which substantial portions of their narratives are true, rather than fabricating an entirely fictitious story. Finally, the datasets employed in this study were collected in experimental low-stake scenarios where participants had low incentives to lie and appear credible. Because of all the above issues, the application of our model in real-life contexts may be limited, and caution is advised when interpreting the results in such situations.

The limitations addressed in this study underscore the need for future research to expand the applicability and generalizability of lie-detection models for real-life settings. Future works may explore the inclusion of new datasets, trying different LLMs (e.g., the most recent GPT-4), different sizes (e.g., FLAN-T5 XXL version), and different fine-tuning strategies to investigate the variance in performance within a lie-detection task. Furthermore, our fine-tuning approach completely erased the previous capabilities possessed by the model; therefore, future works should also focus on new fine-tuning strategies that do not compromise the model’s original capabilities.

## Supplementary Information

Supplementary Information.

## Data Availability

For the Opinion dataset, we obtained full access after contacting the corresponding author. The Memory dataset is downloadable at the link: https://msropendata.com/datasets/0a83fb6f-a759-4a17-aaa2-fbac84577318. The intention dataset is publicly available at the link: https://osf.io/45z7e/.

## Code availabitity

All the Colab Notebooks to perform linguistic analysis on the three datasets, fine-tune the model in the three Scenarios, and conduct explainability analysis is available at https://github.com/robecoder/VerbalLieDetectionWithLLM.git.

## References

[1] Walczyk, J. J., Harris, L. L., Duck, T. K. & Mulay, D. A social-cognitive framework for understanding serious lies: Activation-decision-construction-action theory. *New Ideas Psychol.* **34**, 22–36. 10.1016/j.newideapsych.2014.03.001 (2014).

[2] Amado, B. G., Arce, R. & Fariña, F. Undeutsch hypothesis and criteria based content analysis: A meta-analytic review. *Eur J Psychol Appl Legal Context* **7**, 3–12. 10.1016/j.ejpal.2014.11.002 (2015).

[3] Vrij, A. *et al.* Verbal lie detection: Its past, present and future. *Brain Sciences* **12**, 1644. 10.3390/brainsci12121644 (2022). 36552104 10.3390/brainsci12121644 PMC9775025

[4] Vrij, A. & Fisher, R. P. Which lie detection tools are ready for use in the criminal justice system?. *J. Appl. Res. Mem. Cognit.* **5**, 302–307. 10.1016/j.jarmac.2016.06.014 (2016).

[5] DePaulo, B. M. *et al.* Cues to deception. *Psychol. Bull.* **129**, 74–118. 10.1037/0033-2909.129.1.74 (2003). 12555795 10.1037/0033-2909.129.1.74

[6] Bond, C. F. Jr. & DePaulo, B. M. Accuracy of deception judgments. *Personal. Soc. Psychol. Rev.* **10**, 214–234. 10.1207/s15327957pspr1003_2 (2006). 10.1207/s15327957pspr1003_2 16859438

[7] Levine, T. R., Park, H. S. & McCornack, S. A. Accuracy in detecting truths and lies: Documenting the “veracity effect”. *Commun. Monogr.* **66**, 125–144. 10.1080/03637759909376468 (1999).

[8] Levine, T. R. Truth-default theory (TDT). *J. Lang. Soc. Psychol.* **33,** 378–392. 10.1177/0261927x14535916 (2014).

[9] Street, C. N. H. & Masip, J. The source of the truth bias: Heuristic processing?. *Scand. J. Psychol.* **56**, 254–263. 10.1111/sjop.12204 (2015). 25707774 10.1111/sjop.12204

[10] Verschuere, B., *et al.* The use-the-best heuristic facilitates deception detection. *Nat. Hum. Behav.* **7**, 718–728. 10.1038/s41562-023-01556-2 (2023) 10.1038/s41562-023-01556-2 36941469

[11] Chen, X., Hao, P., Chandramouli, R., and Subbalakshmi, K. P. Authorship similarity detection from email messages. In *International Workshop On Machine Learning and Data Mining In Pattern Recognition*. Editor P. Perner (New York, NY: Springer), 375–386. 10.1007/978-3-642-23199-5_28 (2011).

[12] Chen, H. Dark web: Exploring and mining the dark side of the web. In *2011 European Intelligence and Security Informatics Conference,* 1–2. IEEE (2011).

[13] Daelemans, W. Explanation in computational stylometry. In *Computational Linguistics and Intelligent Text Processing*, 451–462. Springer, Berlin. 10.1007/978-3-642-37256-8_37 (2013).

[14] Hauch, V., Blandón-Gitlin, I., Masip, J. & Sporer, S. L. Are computers effective lie detectors? A meta-analysis of linguistic cues to deception. *Personal. Soc. Psychol. Rev.* **19**, 307–342. 10.1177/1088868314556539 (2015). 10.1177/1088868314556539 25387767

[15] Tomas, F., Dodier, O., & Demarchi, S. Computational measures of deceptive language: Prospects and issues. *Front. Commun.* **7**10.3389/fcomm.2022.792378 (2022).

[16] Conroy, N. K., Rubin, V. L. & Chen, Y. Automatic deception detection: Methods for finding fake news. *Proc. Assoc. Inf. Sci. Technol.* **52**, 1–4. 10.1002/pra2.2015.145052010082 (2015).

[17] Pérez-Rosas, V., Kleinberg, B., Lefevre, A., & Mihalcea, R. Automatic detection of fake news. arXiv preprint arXiv:1708.07104 (2017).

[18] Fornaciari, T. & Poesio, M. Automatic deception detection in Italian court cases. *Artif. Intell. Law* **21**, 303–340. 10.1007/s10506-013-9140-4 (2013).

[19] Yancheva, M., & Rudzicz, F. Automatic detection of deception in child-produced speech using syntactic complexity features. In *Proceedings of the 51st Annual Meeting of the Association for Computational Linguistics* **1,** 944–953, (2013).

[20] Pérez-Rosas, V., & Mihalcea, R. Experiments in open domain deception detection. In *Proceedings of the 2015 Conference on Empirical Methods in Natural Language Processing*. 10.18653/v1/d15-1133 (2015).

[21] Ott, M., Choi, Y., Cardie, C., & Hancock, J. T. Finding deceptive opinion spam by any stretch of the imagination. *arXiv preprint *arXiv:1107.4557 (2011).

[22] Fornaciari, T., & Poesio, M. Identifying fake Amazon reviews as learning from crowds. In *Proceedings of the 14th Conference of the European Chapter of the Association for Computational Linguistics*. 10.3115/v1/e14-1030n (2014).

[23] Kleinberg, B., Mozes, M., Arntz, A. & Verschuere, B. Using named entities for computer-automated verbal deception detection. *Journal of forensic sciences* **63**, 714–723. 10.1111/1556-4029.13645 (2017). 28940300 10.1111/1556-4029.13645

[24] Mbaziira, A. V., & Jones, J. H. Hybrid text-based deception models for native and Non-Native English cybercriminal networks. In *Proceedings of the International Conference on Compute and Data Analysis*. 10.1145/3093241.3093280 (2017).

[25] Levitan, S. I., Maredia, A., & Hirschberg, J. Linguistic cues to deception and perceived deception in interview dialogues. In *Proceedings of the 2018 Conference of the North American Chapter of the Association for Computational Linguistics: Human Language Technologies,* **1.**10.18653/v1/n18-1176 (2018).

[26] Kleinberg, B., Nahari, G., Arntz, A., & Verschuere, B. An investigation on the detectability of deceptive intent about flying through verbal deception detection. *Collabra: Psychol.* **3.**10.1525/collabra.80 (2017).

[27] Constâncio, A. S., Tsunoda, D. F., Silva, H. de F. N., Silveira, J. M. da, & Carvalho, D. R. Deception detection with machine learning: A systematic review and statistical analysis. *PLOS ONE*, **18,** e0281323. 10.1371/journal.pone.0281323 (2023). 10.1371/journal.pone.0281323 PMC9910662 36757928

[28] Zhao, W. X., *et al.* A survey of large language models. *arXiv preprint *arXiv:2303.18223. (2023).

[29] Newman, M. L., Pennebaker, J. W., Berry, D. S. & Richards, J. M. Lying words: Predicting deception from linguistic styles. *Personal. Soc. Psychol. Bull.* **29**, 665–675. 10.1177/0146167203029005010 (2003). 10.1177/0146167203029005010 15272998

[30] Monaro, M. *et al.* Covert lie detection using keyboard dynamics. *Sci Rep* **8**, 1976. 10.1038/s41598-018-20462-6 (2018). 29386583 10.1038/s41598-018-20462-6 PMC5792443

[31] Vrij, A., Fisher, R. P. & Blank, H. A cognitive approach to lie detection: A meta-analysis. *Legal Criminol. Psychol.* **22**(1), 1–21. 10.1111/lcrp.12088 (2015).

[32] Johnson, M. K. & Raye, C. L. Reality monitoring. *Psychol. Rev.* **88**, 67–85. 10.1037/0033-295x.88.1.67 (1981).

[33] Sporer, S. L. The less travelled road to truth: Verbal cues in deception detection in accounts of fabricated and self-experienced events. *Appl. Cognit. Psychol.* **11**(5), 373–397. 10.1002/(SICI)1099-0720(199710)11:5%3c373::AID-ACP461%3e3.0.CO;2-0 (1997).

[34] Sporer, S. L. Reality monitoring and detection of deception in *The Detection of Deception in Forensic Contexts* (Cambridge University Press.)*,* 64–102. 10.1017/cbo9780511490071.004 (2004).

[35] Masip, J., Sporer, S. L., Garrido, E. & Herrero, C. The detection of deception with the reality monitoring approach: A review of the empirical evidence. *Psychol. Crime Law* **11**(1), 99–122. 10.1080/10683160410001726356 (2005).

[36] Amado, B. G., Arce, R., Fariña, F. & Vilariño, M. Criteria-Based Content Analysis (CBCA) reality criteria in adults: A meta-analytic review. *Int. J. Clin. Health Psychol.* **16**(2), 201–210. 10.1016/j.ijchp.2016.01.002 (2016). 30487863 10.1016/j.ijchp.2016.01.002 PMC6225082

[37] Gancedo, Y., Fariña, F., Seijo, D., Vilariño, M. & Arce, R. Reality monitoring: A meta-analytical review for forensic practice. *Eur. J. Psychol. Appl. Legal Context* **13**(2), 99–110. 10.5093/ejpalc2021a10 (2021).

[38] Vrij, A. *et al.* Verbal lie detection: its past, present and future. *Brain Sci.* **12**(12), 1644. 10.3390/brainsci12121644 (2022). 36552104 10.3390/brainsci12121644 PMC9775025

[39] Kleinberg, B., van der Vegt, I., & Arntz, A. Detecting deceptive communication through linguistic concreteness. Center for Open Science. 10.31234/osf.io/p3qjh (2019).

[40] Nahari, G., Vrij, A. & Fisher, R. P. Exploiting liars’ verbal strategies by examining the verifiability of details. *Legal Criminol. Psychol.* **19**, 227–239. 10.1111/j.2044-8333.2012.02069.x (2012).

[41] Vrij, A., & Nahari, G. The verifiability approach. In *Evidence-Based Investigative Interviewing* (pp. 116–133). Routledge. 10.4324/9781315160276-7 (2019).

[42] Pennebaker, J. W., Francis, M. E., & Booth, R. J. Linguistic inquiry and word count: LIWC 2001. *Mahway: Lawrence Erlbaum Associates*, **71,** 2001 (2001).

[43] Boyd, R. L., Ashokkumar, A., Seraj, S., & Pennebaker, J. W. The development and psychometric properties of LIWC-22. Austin, TX: University of Texas at Austin, 1–47. (2022).

[44] Bond, G. D. & Lee, A. Y. Language of lies in prison: Linguistic classification of prisoners’ truthful and deceptive natural language. *Appl. Cognit. Psychol.* **19**(3), 313–329. 10.1002/acp.1087 (2005).

[45] Bond, G. D. *et al.* ‘Lyin’ Ted’, ‘crooked hillary’, and ‘Deceptive Donald’: Language of lies in the 2016 US presidential debates. *Appl. Cognit. Psychol.* **31**(6), 668–677. 10.1002/acp.3376 (2017).

[46] Bond, G. D., Speller, L. F., Cockrell, L. L., Webb, K. G., & Sievers, J. L. ‘Sleepy Joe’ and ‘Donald, king of whoppers’: Reality monitoring and verbal deception in the 2020 U.S. presidential election debates. *Psychol. Rep.* 003329412211052. 10.1177/00332941221105212 (2022). 10.1177/00332941221105212 35634896

[47] Schutte, M., Bogaard, G., Mac Giolla, E., Warmelink, L., Kleinberg, B., & Verschuere, B. *Man versus Machine: Comparing manual with LIWC coding of perceptual and contextual details for verbal lie detection*. Center for Open Science. 10.31234/osf.io/cth58 (2021).

[48] Kleinberg, B., van der Toolen, Y., Vrij, A., Arntz, A. & Verschuere, B. Automated verbal credibility assessment of intentions: The model statement technique and predictive modeling. *Appl. Cognit. Psychol.* **32**, 354–366. 10.1002/acp.3407 (2018). 10.1002/acp.3407 PMC5969289 29861544

[49] Kleinberg, B., & Verschuere, B. How humans impair automated deception detection performance. *Acta Psychol.*, **213**, 10.1016/j.actpsy.2020.103250 (2021). 10.1016/j.actpsy.2020.103250 33450692

[50] Ilias, L., Soldner, F., & Kleinberg, B. Explainable verbal deception detection using transformers. *arXiv preprint *arXiv:2210.03080 (2022).

[51] Capuozzo, P., Lauriola, I., Strapparava, C., Aiolli, F., & Sartori, G. DecOp: A multilingual and multi-domain corpus for detecting deception in typed text. In *Proceedings of the 12th Language Resources and Evaluation Conference*, 1423–1430 (2020).

[52] Sap, M. *et al.* Quantifying the narrative flow of imagined versus autobiographical stories. *Proc. Natl. Acad. Sci.* **119**(45), e2211715119. 10.1073/pnas.2211715119 (2022). 36322749 10.1073/pnas.2211715119 PMC9659415

[53] Hernández-Castañeda, Á., Calvo, H., Gelbukh, A. & Flores, J. J. G. Cross-domain deception detection using support vector networks. *Soft Comput.* **21**, 585–595. 10.1007/s00500-016-2409-2 (2016).

[54] Pérez-Rosas, V., & Mihalcea, R. Cross-cultural deception detection. In *Proceedings of the 52nd Annual Meeting of the Association for Computational Linguistics* **2***.*10.3115/v1/p14-2072 (2014).

[55] Mihalcea, R., & Strapparava, C. The lie detector: Explorations in the automatic recognition of deceptive language. In *Proceedings of the ACL-IJCNLP 2009 conference short papers* 309–312. 10.3115/1667583.1667679 (2009).

[56] Ríssola, E. A., Aliannejadi, M., & Crestani, F. Beyond modelling: Understanding mental disorders in online social media. In *Advances in Information Retrieval: 42nd European Conference on IR Research, ECIR 2020, Lisbon, Portugal, April 14–17, 2020, Proceedings, Part I 42* (pp. 296–310). Springer (2020).

[57] Chung, H. W., *et al.* Scaling instruction-finetuned language models. *arXiv preprint *arXiv:2210.11416. (2022).

[58] Zhou, L., Burgoon, J. K., Nunamaker, J. F. & Twitchell, D. Automating linguistics-based cues for detecting deception in text-based asynchronous computer-mediated communications. *Group Decis. Negot.* **13**, 81–106. 10.1023/b:grup.0000011944.62889.6f (2004).

[59] Solà-Sales, S., Alzetta, C., Moret-Tatay, C. & Dell’Orletta, F. Analysing deception in witness memory through linguistic styles in spontaneous language. *Brain Sci.* **13**, 317. 10.3390/brainsci13020317 (2023). 36831859 10.3390/brainsci13020317 PMC9953826

[60] Sarzynska-Wawer, J., Pawlak, A., Szymanowska, J., Hanusz, K. & Wawer, A. Truth or lie: Exploring the language of deception. *PLOS ONE* **18**, e0281179. 10.1371/journal.pone.0281179 (2023). 36730363 10.1371/journal.pone.0281179 PMC9894434

[61] Brysbaert, M., Warriner, A. B. & Kuperman, V. Concreteness ratings for 40 thousand generally known English word lemmas. *Behav Res* **46**, 904–911. 10.3758/s13428-013-0403-5 (2014). 10.3758/s13428-013-0403-5 24142837

[62] Lin, Y. C., Chen, S. A., Liu, J. J., & Lin, C. J. Linear Classifier: An Often-Forgotten Baseline for Text Classification. *arXiv preprint *arXiv:2306.07111 (2023).

[63] Moore, J. H. Bootstrapping, permutation testing and the method of surrogate data. *Phys. Med. Biol.* **44**(6), L11 (1999). 10498505 10.1088/0031-9155/44/6/101

[64] McGraw, K. O. & Wong, S. P. A common language effect size statistic. *Psychol. Bull.* **111**, 361. 10.1037/0033-2909.111.2.361 (1992).

[65] Hancock, J. T., Curry, L. E., Goorha, S. & Woodworth, M. On lying and being lied to: A linguistic analysis of deception in computer-mediated communication. *Discourse Process.* **45**, 1–23. 10.1080/01638530701739181 (2007).

