# Supplementary material for: Verbal lie detection using Large Language Models
Source: Sci Rep. 2023 Dec 21;13:22849. doi: 10.1038/s41598-023-50214-0 (PMC10739834; doi:10.1038/s41598-023-50214-0)
Supplement: Supplementary file 1 — Supplementary Information. [file 41598_2023_50214_MOESM1_ESM.pdf]

Supplementary Information

Verbal Lie Detection using Large Language Models

Riccardo Loconte [1*], Roberto Russo [2], Pasquale Capuozzo[3], Pietro Pietrini [1], Giuseppe Sartori [3]

Email addresses: riccardo.loconte@imtlucca.it (R. Loconte)*, roberto.russo.4@studenti.unipd.it (R. Russo), pasqualecapuozzo@gaslini.org (P. Capuozzo), pietro.pietrini@imtlucca.it (P. Pietrini), giuseppe.sartori@unipd.it (G. Sartori)

[1] Molecular Mind Lab, IMT School of Advanced Studies Lucca, Lucca, Italy

[2] Department of Mathematics "Tullio Levi-Civita", University of Padova, Padova, Italy

[3] Department of General Psychology, University of Padova, Padova, Italy

* Corresponding author. IMT School of Advanced Studies Lucca, Piazza San Francesco 19, Lucca (LU) 55100, Italy. Email: riccardo.loconte@imtlucca.it

Supplementary Material: Dataset description

The **Deceptive Opinions dataset** [1] consists of 5000 opinions about highly controversial issues such as abortion, cannabis legalization, gay marriage, euthanasia, and policy on migrants. The dataset was collected from two samples in the US and Italy through Amazon Mechanical Turk, and participants were asked to provide both truthful and deceptive opinions in their native language (i.e., English or Italian). The experimental paradigm employed a ground-truth approach in which participants were instructed to provide both truthful and deceptive opinions for half of the topics, ensuring counterbalancing of the proportion of truthful and deceptive opinions for each topic. For our study, we selected opinions collected from the English (US) sample for a total of 2500 opinions from 500 participants.

The **Hippocorpus dataset** [2] is a collection of stories gathered through three stages on Amazon Mechanical Turk. The first stage involved workers writing a story about a memorable event they experienced in the past six months. The second stage involved workers from the first stage retelling their stories after 3-6 months. In the third stage, new workers were assigned a subset of story topics from the first stage and instructed to imagine a complete narrative based on that topic. They were then asked to write down the story as if they had personally experienced it. After writing, workers completed a questionnaire about the personal significance of their stories. The dataset contains 6,854 stories: 2,779 recalled stories, 2,756 imagined stories, and 1,319 retold stories. For the aim of this study, we included only recalled and imagined stories. Additionally, 11 stories were excluded due to missing data, and 19 stories containing a number of words below 2.5 standard deviations from the average (narratives with less than 24.34 words) were removed. We considered these stories to have too few words for analysis and indicative of a lack of engagement among the Turkers. Therefore, the final sample of stories employed for the study was 5,506.

The **Intention dataset** [3] is a collection of statements about participants' most significant non-work-related activity recruited via Prolific Academic. Participants were instructed to provide convincing answers to two brief questions:

- Q1. "Please describe your activity as specific as possible";
- Q2. "Which information can you give us to reassure us that you are telling the truth?".

The activity described was required to be specific and not a continuous or daily activity occurring within the next seven days, with a defined start and end time.
Participants were randomly assigned to either the truthful or deceptive condition. In the deceptive condition, participants were assigned matched activities from the truthful condition. The dataset contains 1640 statements (857 deceptive and 783 truthful) with two answers per participant. For the aim of this study, we selected only statements from Q1, for a total of 1640 statements.

Supplementary Material: Vocabulary Uniqueness

Vocabulary uniqueness was computed by applying the Jaccard's index to the truthful and deceptive vocabulary sets for each dataset, as in Ríssola et al., 2020 and Ilias et al., 2022 [4,5]. Jaccard's index is a measure of **similarity between two sample sets**. In the context of text analysis, we used the Jaccard's index to compare the vocabulary used in truthful and deceptive statements as in the following equation:

$$J = (setTrue \cap setFalse) \,/\, (setTrue \cup setFalse) \quad (1)$$

To ensure the accuracy of our Jaccard's index calculations, we implemented a series of preprocessing steps on the text data using the Python library SpaCy. These steps included converting all text to lowercase, applying tokenization to segment the text into individual words, removing stop words, and lemmatizing the remaining words. This preprocessing was necessary to focus solely on content words when computing the Jaccard's index.

Subsequently, we transformed the preprocessed text samples for each group (truthful vs. deceptive) into sets of unique words utilizing the `split()` and `explode()` methods available in the Pandas library. This approach allowed us to construct sets comprising all the distinct words present in the truthful and deceptive text samples. The Jaccard's index was derived by calculating the intersection (common words) and union (total words) of these two sets. The resulting index ranges from 0, indicating a completely different vocabulary between the two sets, to 1, indicating a completely identical vocabulary between the two sets. This index served as a measure of similarity or overlap between the word choices of truthful and deceptive statements within the respective datasets.

Supplementary Material: DeCLaRatiVE STYLOMETRY

Using several Natural Language Processing (NLP) techniques, we computed **D**e**CL**a**R**ati**VE** stylometry to a) describe linguistic differences in truthful and deceptive statements in the three datasets and b) conduct explainability analysis by exploring the linguistic style of sentences the model correctly classified and misclassified in order to understand whether the style of those sentences was a relevant feature for the model to generate its predictions. **D**e**CL**a**R**ati**VE** stylometry consisted of the extraction process of 26 linguistic features among the psychological frameworks of **D**istancing [6], **C**ognitive **L**oad [7], **R**eality **M**onitoring [8], and **VE**rifiability Approach [9,10]. The extraction process for each framework is well described in the paragraphs below.

1. COGNITIVE LOAD:

Previous research has associated statistics about length and readability of the text to complexity and were employed to study deception along with the cognitive load framework [11,12,13,14] (see also [15] for a metanalysis of studies of linguistic cues for deception).

Using the Python library TEXTSTAT, we automatically computed several statistics from raw texts:

- number of sentences (**num_sentences**),
- number of words (**num_words**),
- number of syllables (**num_syllables**),
- the average number of syllables per word (**avg_syllabes_per_word**),
- the Flesch-Kincaid Grade Level (**fk_grade**),
- the Flesch Reading-Ease Level (**fk_read**)

The Flesch-Kincaid Grade Level and the Flesch Reading-Ease Level were used to determine the difficulty of understanding a passage in English. Both tests utilize the same basic measures of word length and sentence length but differ in their weighting factors.
The results of these tests are inversely correlated: a text with a high score on the Reading Ease test will typically have a lower score on the Grade-Level test. Higher scores on the Flesch Reading-Ease test indicate that the material is easier to read, while lower scores indicate more difficult passages. The Flesch reading-ease score is computed as in equation (1) [16]:

$$(1)206.835 - 1.015(total\ words/total\ sentences) - 84.6(total\ syllables/total\ words)$$

The Flesch-Kincaid Grade Level Formula produces a score corresponding to a U.S. grade level, providing an intuitive index about the readability level of texts. The index may be interpreted as the years of education typically required to understand the text. The grade level is computed as in equation (2) [17]:

$$0.39(total\ words/total\ sentences) + 11.8(total\ syllables/total\ words) - 15.59 \quad (2)$$

2. REALITY MONITORING AND DISTANCING FRAMEWORK

2.1 LINGUISTIC INQUIRY AND WORD COUNT (LIWC)

Linguistic Inquiry and Word Count (LIWC) is the gold standard software for analyzing word usage [18]. Given a text, it calculates the percentage of total words corresponding to more than 100 categories in the dictionary related to different psychosocial dimensions, which have been validated by human evaluators using rigorous procedures. A detailed description of LIWC-22 functioning and categories is reported in [19]. Using the English dictionary, we scored each text along with all the categories present in LIWC-22.

LIWC scoring was computed on tokenized text using the English dictionary. The selection of the LIWC categories was guided by previous research on computerized verbal lie-detection [2, 6] and a recent metanalysis [15]. Therefore, we employed LIWC to investigate the presence of verbal cues related to the Reality Monitoring and Distancing frameworks. Additionally, we computed summary scores about the analytical style, the authenticity, and the tone of the text, as well as indices about the writer's temporal orientation.

The selected and compounded linguistic features of interest are listed below:

- Summary statistics:
  - **'Analytic':** It describes the degree to which people use words that suggest formal, logical, and hierarchical thinking patterns, as known as Analytic Thinking. People low in Analytical Thinking tend to write and think using more intuitive and personal language. Language scoring high in Analytic Thinking tends to be rewarded in academic settings and correlates with grades and reasoning skills. Language scoring low in Analytic Thinking tends to be viewed as less cold and rigid and more friendly and personable.
  - **'Authentic':** It describes the degree to which a person is self-monitoring while speaking or writing. Examples of texts that score low in authenticity include prepared texts (i.e., speeches written ahead of time) and texts where a person is being socially cautious. Examples of highly authentic texts tend to be spontaneous conversations between close friends or political leaders with little-to-no social inhibitions.
  - **'Tone':** Although LIWC-22 includes both positive and negative tone dimensions, the Tone variable puts the two dimensions into a single summary variable. The algorithm is built so that the more positive the tone, the higher the score. Scores below 50 suggest a more negative emotional tone.
- Basic Dictionary:
  - **'tone_pos':** provides the percentage score of words related to a positive sentiment (rather than positive emotion per se). It includes words related to positive emotions (e.g., happy, joy) and words related to those emotions (e.g., birthday, beautiful).
  - **'tone_neg':** provides the percentage score of words related to a negative sentiment

  (rather than negative emotion per se). It includes words related to negative emotions (e.g., sad, angry) and words related to those (e.g., kill, funeral).
  - **'Cognition':** It is the overarching dimension that reflects different ways people think or refer to their thinking. It includes the subcategories of all-or-none thinking, different cognitive processes (i.e., insight, causation, discrepancy, tentative, certitude, differentiation), and memory.
    - **'memory'**: (e.g., remember, forget) reflect people's references and attention to their memories, beliefs about memory, and the processes of recall and forgetting.

- Writer's temporal orientation:
  - **'focuspast'**: refers to the use of past tense verbs and adverbs related to the past in language.
  - **'focuspresent'**: refers to the use of present tense verbs and adverbs related to the present in language.
  - **'focusfuture'**: refers to the use of future tense verbs and adverbs related to the future in language.

The following scores were computed by feature-engineering the available LIWC features according to theories on lie detection:

- To investigate the use of personal pronouns along with the **Distancing framework,** we computed two metrics [6]:

  - **Self-reference:** computed as the sum of LIWC categories: 'i' + 'we'
  - **Other-reference:** computed as the sum of LIWC categories: 'shehe' + 'they' + 'you'

- To investigate the **Reality Monitoring framework**, we computed the following metrics below, following the same procedure as in [20,21,22,23]:

  - **Contextual Embedding**: computed as the sum of LIWC categories: 'space' + 'motion' + 'time'
  - **Perceptual Details:** computed as the sum of LIWC categories: 'attention' + 'visual' + 'auditory' + 'feeling'
  - **Reality Monitoring:** computed as the sum of LIWC categories: Contextual Embedding + Perceptual Details + 'Affect' - 'Cognition'

2.2 CONCRETENESS SCORE

Another way to investigate the assumption of Reality Monitoring is through linguistic concreteness of words used in truthful and deceptive texts. Kleinberg et al. (2019) [24] have already investigated the concreteness of words in the framework of lie detection postulating the *truthful concreteness hypothesis*, which states that truthful accounts are typically characterized by specific, concrete,

and situationally embedded information, while deceptive and fabricated statements tend to contain more abstract and less concrete information.

To determine the average level of concreteness for each statement, we utilized the concreteness annotation dataset developed by Brysbaert et al. (2014) [25] (download is available in the Supplementary Materials here: https://link.springer.com/article/10.3758/s13428-013-0403-5#Sec10). This dataset involved around 40.000 English word lemmas, which were scored by a large group of human annotators using a five-point Likert scale, ranging from 1 (abstract) to 5 (concrete).

As for the computation of the Jaccard's index, we implemented a series of preprocessing steps on the text data using the Python library SpaCy to ensure the accuracy of our ***concreteness score*** calculation. These steps included converting all text to lowercase, applying tokenization to segment the text into individual words, removing stop words, and lemmatizing the remaining words. This process resulted in a list of lemmatized content words. Lemmatization was a necessary step to ensure a consistent overlap between our list of content words and those in the annotated concreteness dataset.

To compute the average concreteness score for each text, we cross-referenced the content words with the annotated concreteness dataset, assigning the respective concreteness scores when a match was found. Finally, we computed our dependent variable '`concr_score`' by averaging the concreteness scores of all content words in the list. Higher values of the '`concr_score`' indicated a greater degree of concreteness in the language employed within the statements.

3. VERIFIABILITY APPROACH

The verifiability approach in verbal lie detection suggests that truthful statements are more likely to be verifiable than false or deceptive statements, as liars avoid mentioning details that could be verified with independent evidence to conceal their deception [9, 10]. Verifiable details may be represented by activities involving or witnessed by identified individuals, documented through video or photographic evidence, or leaving digital or physical traces (e.g., phone calls or receipts) [9, 10].

Automatically extracting **verifiable details** by using **named-entity recognition** (NER) has been proven to be effective for the detection of deception in hotel reviews [26] as well as in participants' intentions on their weekend plans [27].

Named Entity Recognition (NER) is a Natural Language Processing (NLP) technique that deals with identifying and extracting information from text (so-called named entities) and classifying them into predefined categories, such as persons, locations, organizations, time, and many more Using SpaCy, a Python library for NLP, we automatically extracted unique named entities from each raw statement through a Transformer algorithm for English language (*`en_core_web_trf`*, https://spacy.io/models/en#en_core_web_trf).

Supplementary Table S3 shows the list of all entities available in SpaCy with their descriptions and some examples. Supplementary Table S4 shows the list of the combined entities along with the Verifiability approach.

Entity	Description	Example
DATE	Absolute or relative dates or periods	“December 25, 2022”, “10th August 1998”, “Yesterday”
TIME	Times smaller than a day	“2:30 PM”, “9 o’clock”, “morning”
GPE	Countries, cities, states	“United States”, “Paris”, “Tokyo”
LOC	Non-GPE locations, mountain ranges, bodies of water	“Central Park”, “Mount Everest”, “Amazon Rainforest”
PERSON	People, including fictional	“Steve Jobs”, “Emma Johnson”, “Harry Potter”
ORDINAL	“first”, “second”, etc.	“First”, “Third”, “Tenth”
ORG	Companies, agencies, institutions, etc.	“Google”, “Apple Inc.”, “United Nations”
QUANTITY	Measurements, as of weight or distance	“10 kilograms”, “5 liters”, “100 meters”
WORK_OF_ART	Titles of books, songs, etc.	“Mona Lisa”, “Hamlet”, “Gone with the Wind”
PRODUCT	Objects, vehicles, foods, etc. (not services)	“iPhone”, “Coca-Cola”, “Nike shoes”
CARDINAL	Numerals that do not fall under another type	“Five”, “Twenty”, “One hundred”
NORP	Nationalities or religious or political groups	“American”, “Muslim”, “Republican”
MONEY	Monetary values, including unit	“$10”, “€50”, “¥1000”
LANGUAGE	Any named language	“English”, “Spanish”, “French”
FAC	Buildings, airports, highways, bridges, etc.	“Eiffel Tower”, “White House”, “Golden Gate Bridge”
EVENT	Named hurricanes, battles, wars, sports events, etc.	“Olympic Games”, “Wedding ceremony”, “Concert”
PERCENT	Percentage, including “%”	“50%”, “10.5%”, “75.2%”
LAW	Named documents made into laws.	“Constitution”, “Copyright Act”, “Traffic laws”

Supplementary Table S3. List of the labels, brief descriptions, and few examples of the extracted named-entities with the Python library SpaCy.

Label	Grouped Entities
**People**	PERSON
**Temporal details**	DATE + TIME + EVENT
**Spatial details**	GPE + LOC + FAC
**Quantity details**	PERCENT + MONEY + QUANTITY + CARDINAL + ORDINAL

Supplementary Table S4. New linguistic features derived after grouping named-entities.

Truthful statements are expected to include more named-entities because they are typically richer in detail [8, 28], are characterized by more verifiable details [29], and are often more contextualized in time and space [30]. The example depicted in Supplementary Figure S1 is a common way to represent text with annotated named-entities.

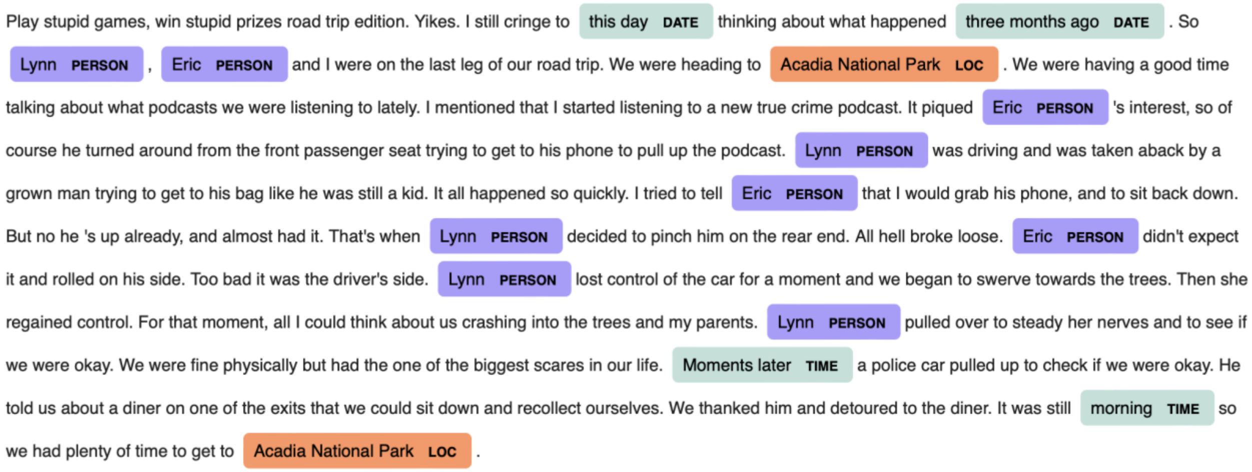

Supplementary Figure S1. Narrative of an autobiographical event annotated with named entities using Named-Entities Recognition in SpaCy. In this narrative, entities related to named people (e.g., Lynn and Eric), location (e.g., Acadia National Park), dates (e.g., this day, three months ago), and times (e.g., moments later, morning) were automatically detected.

Supplementary Results: Descriptive Linguistic Analysis

In Table S5 we provided the results of the descriptive linguistic analysis conducted on the three datasets using the **D**e**CL**a**R**ati**VE** stylometry.

Linguistic Feature	Dataset	Permutation t-statistic	Corrected p-value	CLES (95 % CI)	Cohen's D (95% CI)	Direction
num_sentences	Opinion	0.264	0.006**	0.57 (0.55, 0.59)	0.19 (0.1, 0.28)	Truthful
	Memory	0.138	0.365	0.52 (0.5, 0.53)	0.04 (-0.02, 0.09)	Truthful
	Intention	-0.092	1	0.47 (0.44, 0.5)	-0.06 (-0.15, 0.04)	Deceptive
word_counts	Opinion	15.508	0.005**	0.68 (0.66, 0.7)	0.57 (0.48, 0.67)	Truthful
	Memory	29.268	0.005**	0.6 (0.59, 0.62)	0.32 (0.27, 0.37)	Truthful
	Intention	-6.509	0.005**	0.43 (0.4, 0.46)	-0.22 (-0.31, -0.12)	Deceptive
num_syllables	Opinion	23.182	0.005**	0.68 (0.66, 0.7)	0.61 (0.51, 0.7)	Truthful
	Memory	41.277	0.005**	0.61 (0.6, 0.62)	0.35 (0.3, 0.41)	Truthful
	Intention	-7.5	0.005**	0.44 (0.41, 0.47)	-0.20 (-0.3, -0.1)	Deceptive
avg_syllabes_per_word	Opinion	0.027	0.005**	0.58 (0.55, 0.6)	0.27 (0.18, 0.36)	Truthful
	Memory	0.015	0.005**	0.56 (0.54, 0.57)	0.21 (0.16, 0.27)	Truthful
	Intention	0.003	1	0.50 (0.47, 0.53)	0.03 (-0.07, 0.13)	Truthful
fk_grade	Opinion	1.395	0.005**	0.67 (0.65, 0.69)	0.56 (0.47, 0.66)	Truthful
	Memory	0.863	0.005**	0.63 (0.61, 0.64)	0.46 (0.4, 0.51)	Truthful
	Intention	-0.465	0.094	0.46 (0.43, 0.49)	-0.13 (-0.23, -0.03)	Deceptive
	Opinion	-5.095	0.005**	0.37 (0.34, 0.39)	-0.48 (-0.57, -0.39)	Deceptive

Linguistic Feature	Dataset	Permutation t-statistic	Corrected p-value	CLES (95 % CI)	Cohen's D (95% CI)	Direction
fk_read	Memory	-3.133	0.005**	0.39 (0.37, 0.4)	-0.39 (-0.45, -0.34)	Deceptive
	Intention	0.984	1	0.52 (0.49, 0.55)	0.08 (-0.02, 0.17)	Truthful
Analytic	Opinion	0.624	0.623	0.50 (0.47, 0.53)	0.03 (-0.06, 0.12)	Truthful
	Memory	5.139	0.005**	0.57 (0.55, 0.58)	0.23 (0.18, 0.29)	Truthful
	Intention	5.859	0.005**	0.57 (0.54, 0.59)	0.21 (0.12, 0.31)	Truthful
Authentic	Opinion	14.984	0.005**	0.69 (0.67, 0.72)	0.70 (0.6, 0.8)	Truthful
	Memory	0.605	0.415	0.5 (0.49, 0.52)	0.02 (-0.03, 0.07)	Truthful
	Intention	3.329	0.449	0.54 (0.51, 0.56)	0.10 (0.01, 0.2)	Truthful
Tone	Opinion	8.191	0.005**	0.63 (0.59, 0.66)	0.41 (0.32, 0.51)	Truthful
	Memory	-2.545	0.015*	0.48 (0.46, 0.49)	-0.08 (-0.13, -0.03)	Deceptive
	Intention	-4.505	0.073	0.47 (0.44, 0.49)	-0.14 (-0.24, -0.05)	Deceptive
tone_pos	Opinion	0.633	0.005**	0.62 (0.58, 0.65)	0.37 (0.28, 0.46)	Truthful
	Memory	-0.334	0.005**	0.45 (0.44, 0.47)	-0.16 (-0.21, -0.1)	Deceptive
	Intention	-0.307	0.664	0.46 (0.44, 0.49)	-0.09 (-0.19, 0.01)	Deceptive
tone_neg	Opinion	-1.138	0.005**	0.38 (0.34, 0.41)	-0.42 (-0.51, -0.33)	Deceptive
	Memory	-0.1	0.029*	0.48 (0.47, 0.49)	-0.08 (-0.13, -0.02)	Deceptive
	Intention	-0.01	1	0.48 (0.47, 0.5)	-0.01 (-0.11, 0.09)	Deceptive
Cognition	Opinion	1.201	0.005**	0.59 (0.55, 0.62)	0.26 (0.17, 0.35)	Truthful
	Memory	-0.991	0.005**	0.43 (0.41, 0.44)	-0.27 (-0.32, -0.22)	Deceptive

**Linguistic Feature**	**Dataset**	**Permutation t-statistic**	**Corrected p-value**	**CLES (95 % CI)**	**Cohen's D (95% CI)**	**Direction**
	Intention	-1.175	0.005**	0.42 (0.39, 0.45)	-0.25 (-0.35, -0.16)	Deceptive
memory	Opinion	-0.003	0.595	0.50 (0.49, 0.51)	-0.03 (-0.12, 0.05)	Deceptive
	Memory	0.054	0.005**	0.54 (0.53, 0.55)	0.12 (0.07, 0.17)	Truthful
	Intention	-0.013	1	0.50 (0.49, 0.5)	-0.05 (-0.15, 0.05)	Deceptive
focuspast	Opinion	0.23	0.048*	0.56 (0.52, 0.59)	0.18 (0.09, 0.27)	Truthful
	Memory	0.213	0.064	0.51 (0.5, 0.53)	0.07 (0.01, 0.12)	Truthful
	Intention	-0.835	0.005**	0.40 (0.38, 0.43)	-0.36 (-0.46, -0.26)	Deceptive
focuspresent	Opinion	-0.489	0.029*	0.45 (0.42, 0.48)	-0.20 (-0.29, -0.11)	Deceptive
	Memory	-0.226	0.005**	0.46 (0.45, 0.48)	-0.13 (-0.18, -0.08)	Deceptive
	Intention	-0.605	0.005**	0.44 (0.41, 0.46)	-0.20 (-0.3, -0.1)	Deceptive
focusfuture	Opinion	-0.264	0.008**	0.46 (0.43, 0.49)	-0.21 (-0.3, -0.12)	Deceptive
	Memory	-0.228	0.005**	0.45 (0.44, 0.47)	-0.2 (-0.25, -0.14)	Deceptive
	Intention	0.107	1	0.51 (0.48, 0.54)	0.03 (-0.07, 0.12)	Truthful
Self-reference	Opinion	1.052	0.005**	0.62 (0.59, 0.65)	0.41 (0.32, 0.5)	Truthful
	Memory	-0.536	0.005**	0.44 (0.43, 0.46)	-0.18 (-0.23, -0.13)	Deceptive
	Intention	-0.572	0.039*	0.45 (0.42, 0.48)	-0.15 (-0.25, -0.05)	Deceptive
Other-reference	Opinion	-0.654	0.005**	0.43 (0.39, 0.46)	-0.29 (-0.38, -0.2)	Deceptive
	Memory	-0.128	0.342	0.49 (0.47, 0.5)	-0.04 (-0.1, 0.01)	Deceptive
	Intention	-0.42	0.048*	0.45 (0.43, 0.48)	-0.14 (-0.24, -0.05)	Deceptive

Linguistic Feature	Dataset	Permutation t-statistic	Corrected p-value	CLES (95 % CI)	Cohen's D (95% CI)	Direction
Perceptual details	Opinion	0.469	0.005**	0.65 (0.62, 0.69)	0.45 (0.36, 0.54)	Truthful
	Memory	-0.089	0.083	0.48 (0.47, 0.5)	-0.06 (-0.11, -0.01)	Deceptive
	Intention	-0.203	1	0.46 (0.44, 0.49)	-0.07 (-0.17, -0.03)	Deceptive
Contextual Embedding	Opinion	-0.736	0.007**	0.44 (0.4, 0.47)	-0.22 (-0.31, -0.13)	Deceptive
	Memory	0.729	0.005**	0.55 (0.54, 0.57)	0.19 (0.14, 0.24)	Truthful
	Intention	1.84	0.005**	0.56 (0.54, 0.59)	0.24 (0.15, 0.34)	Truthful
Reality Monitoring	Opinion	-1.994	0.005**	0.42 (0.38, 0.45)	-0.27 (-0.36, -0.18)	Deceptive
	Memory	1.217	0.005**	0.55 (0.54, 0.57)	0.2 (0.14, 0.25)	Truthful
	Intention	2.474	0.005**	0.58 (0.55, 0.6)	0.26 (0.17, 0.36)	Truthful
Concreteness score	Opinion	-0.112	0.005**	0.32 (0.29, 0.35)	-0.60 (-0.69, -0.5)	Deceptive
	Memory	0.037	0.005**	0.54 (0.53, 0.56)	0.17 (0.11, 0.22)	Truthful
	Intention	0.125	0.005**	0.61 (0.59, 0.64)	0.39 (0.29, 0.49)	Truthful
People	Opinion	-0.038	0.111	0.49 (0.48, 0.51)	-0.10 (-0.19, -0.01)	Deceptive
	Memory	-0.001	0.005**	0.45 (0.44, 0.46)	-0.23 (-0.28, -0.17)	Deceptive
	Intention	-0.335	0.005**	0.45 (0.43, 0.47)	-0.24 (-0.34, -0.14)	Deceptive
Temporal details	Opinion	0.011	0.663	0.52 (0.5, 0.55)	0.03 (-0.06, 0.12)	Truthful
	Memory	0.002	0.005**	0.56 (0.54, 0.57)	0.2 (0.15, 0.25)	Truthful
	Intention	0.105	1	0.50 (0.47, 0.53)	0.03 (-0.06, 0.13)	Truthful
	Opinion	0.003	0.934	0.51 (0.49, 0.54)	0.01 (-0.08, 0.09)	Truthful

Linguistic Feature	Dataset	Permutation t-statistic	Corrected p-value	CLES (95 % CI)	Cohen's D (95% CI)	Direction
Spatial details	Memory	0.001	0.005**	0.53 (0.51, 0.54)	0.09 (0.04, 0.15)	Truthful
	Intention	-0.096	1	0.47 (0.45, 0.49)	-0.05 (-0.15, 0.04)	Deceptive
Quantity details	Opinion	0.022	0.448	0.52 (0.5, 0.55)	0.05 (-0.04, 0.14)	Truthful
	Memory	0.002	0.005**	0.58 (0.56, 0.59)	0.29 (0.23, 0.34)	Truthful
	Intention	0.352	0.005**	0.53 (0.51, 0.55)	0.22 (0.12, 0.31)	Truthful

Table S5. Descriptive linguistic analysis from the DeCLaRatiVE stylometry for each dataset (Opinion, Memory, Intention), statistic of the permutation t-test, p-values after Holm-Bonferroni correction (* $p < .05$, ** $p < .01$, *** $p < .001$), effect size (Common language effect size and Cohen's D) with 95 % confidence intervals, and direction of the effect (truthful vs. deceptive).

Supplementary References

[1] Capuozzo, P., Lauriola, I., Strapparava, C., Aiolli, F., & Sartori, G. DecOp: A multilingual and multi-domain corpus for detecting deception in typed text. In *Proceedings of the 12th Language Resources and Evaluation Conference* (pp. 1423-1430). (2020, May).

[2] Sap, M., Horvitz, E., Choi, Y., Smith, N. A., & Pennebaker, J. Recollection versus imagination: Exploring human memory and cognition via neural language models. In *Proceedings of the 58th annual meeting of the association for computational linguistics* (pp. 1970-1978). http://dx.doi.org/10.18653/v1/2020.acl-main.178 (2020, July).

[3] Kleinberg, B., & Verschuere, B. How humans impair automated deception detection performance. *Acta Psychologica*, **213**, 103250. https://doi.org/10.1016/j.actpsy.2020.103250 (2021).

[4] Ríssola, E. A., Aliannejadi, M., & Crestani, F. Beyond modelling: Understanding mental disorders in online social media. In *Advances in Information Retrieval: 42nd European Conference on IR Research, ECIR 2020, Lisbon, Portugal, April 14–17, 2020, Proceedings, Part I 42* (pp. 296-310). Springer International Publishing. (2020).

[5] Ilias, L., Soldner, F., & Kleinberg, B. Explainable Verbal Deception Detection using Transformers. *arXiv preprint arXiv:2210.03080*. (2022).

[6] Newman, M. L., Pennebaker, J. W., Berry, D. S., & Richards, J. M. (2003). Lying words: Predicting deception from linguistic styles. *Personality and Social Psychology Bulletin*, *29*(5), 665–675. https://doi.org/10.1177/0146167203029005010

[7] Vrij, A., Fisher, R., Mann, S., & Leal, S. A cognitive load approach to lie detection. *Journal of Investigative Psychology and Offender Profiling*, **5,** 39–43. https://doi.org/10.1002/jip.82 (2008).

[8] Johnson, M. K., & Raye, C. L. Reality monitoring. *Psychological Review*, **88,** 67–85. https://doi.org/10.1037/0033-295x.88.1.67 (1981).

[9] Nahari, G., Vrij, A., & Fisher, R. P. Exploiting liars' verbal strategies by examining the verifiability of details. *Legal and Criminological Psychology*, **19,** 227–239. https://doi.org/10.1111/j.2044-8333.2012.02069.x (2012).

[10] Vrij, A., & Nahari, G. The verifiability approach. In *Evidence-Based Investigative Interviewing* (pp. 116–133). Routledge. http://dx.doi.org/10.4324/9781315160276-7 (2019).

[11] Zhou, L., Burgoon, J. K., Nunamaker, J. F., & Twitchell, D. Automating linguistics-based cues for detecting deception in text-based asynchronous computer-mediated communications. *Group Decision and Negotiation*, **13,** 81–106. https://doi.org/10.1023/b:grup.0000011944.62889.6f (2004).

[12] Pérez-Rosas, V., & Mihalcea, R. Experiments in open domain deception detection. *Proceedings of the 2015 Conference on Empirical Methods in Natural Language Processing*. http://dx.doi.org/10.18653/v1/d15-1133 (2015).

[13] Solà-Sales, S., Alzetta, C., Moret-Tatay, C., & Dell'Orletta, F. Analysing deception in witness memory through linguistic styles in spontaneous language. *Brain Sciences*, **13,** 317. https://doi.org/10.3390/brainsci13020317 (2023).

[14] Sarzynska-Wawer, J., Pawlak, A., Szymanowska, J., Hanusz, K., & Wawer, A. Truth or lie: Exploring the language of deception. *PLOS ONE*, **18,** e0281179. https://doi.org/10.1371/journal.pone.0281179 (2023).

[15] Hauch, V., Blandón-Gitlin, I., Masip, J., & Sporer, S. L. Are computers effective lie detectors? A meta-analysis of linguistic cues to deception. *Personality and social psychology Review*, **19,** 307-342. https://doi.org/10.1177/1088868314556539 (2015).

[16] Flesch, R. How to write plain English. *University of Canterbury. Available at http://www. mang. canterbury. ac. nz/writing_guide/writing/flesch.shtml. [Retrieved 5 February 2016]*. (1979)

[17] Kincaid, J. P., Fishburne Jr, R. P., Rogers, R. L., & Chissom, B. S. Derivation of new readability formulas (automated readability index, fog count and flesch reading ease formula) for navy enlisted personnel. *Naval Technical Training Command Millington TN Research Branch*. (1975).

[18] Pennebaker, J. W., Francis, M. E., & Booth, R. J. Linguistic inquiry and word count: LIWC 2001. *Mahway: Lawrence Erlbaum Associates*, **71,** 2001 (2001).

[19] Boyd, R. L., Ashokkumar, A., Seraj, S., & Pennebaker, J. W. The development and psychometric properties of LIWC-22. Austin, TX: University of Texas at Austin, 1-47. (2022).

[20] Bond, G. D., & Lee, A. Y. Language of lies in prison: Linguistic classification of prisoners' truthful and deceptive natural language. *Applied Cognitive Psychology*, **19,** 313–329. https://doi.org/10.1002/acp.1087 (2005).

[21] Bond, G. D., *et al.* 'Lyin' Ted', 'crooked hillary', and 'Deceptive Donald': Language of lies in the 2016 US presidential debates. *Applied Cognitive Psychology*, **31,** 668–677. https://doi.org/10.1002/acp.3376 (2017).

[22] Kleinberg, B., Nahari, G., Arntz, A., & Verschuere, B. An investigation on the detectability of deceptive intent about flying through verbal deception detection. *Collabra: Psychology*, **3,** https://doi.org/10.1525/collabra.80 (2017).

[23] Bond, G. D., Speller, L. F., Cockrell, L. L., Webb, K. G., & Sievers, J. L. 'Sleepy Joe' and 'Donald, King of Whoppers': Reality monitoring and verbal deception in the 2020 U.S. presidential election debates. *Psychological Reports*, **0** https://doi.org/10.1177/00332941221105212 (2022)

[24] Kleinberg, B., van der Vegt, I., & Arntz, A. Detecting deceptive communication through linguistic concreteness. Center for Open Science. http://dx.doi.org/10.31234/osf.io/p3qjh (2019).

[25] Brysbaert, M., Warriner, A.B. & Kuperman, V. Concreteness ratings for 40 thousand generally known English word lemmas. *Behav Res* **46**, 904–911 https://doi.org/10.3758/s13428-013-0403-5 (2014).

[26] Kleinberg, B., Mozes, M., Arntz, A., & Verschuere, B. Using named entities for computer-automated verbal deception detection. *Journal of forensic sciences*, **63**, 714-723. https://doi.org/10.1111/1556-4029.13645 (2017).

[27] Kleinberg, B., van der Toolen, Y., Vrij, A., Arntz, A., & Verschuere, B. Automated verbal credibility assessment of intentions: The model statement technique and predictive modeling. *Applied Cognitive Psychology*, **32**, 354–366. https://doi.org/10.1002/acp.3407 (2018).

[28] Masip, J., Sporer, S. L., Garrido, E., & Herrero, C. The detection of deception with the reality monitoring approach: A review of the empirical evidence. *Psychology, Crime & Law*, **11**, 99–122. https://doi.org/10.1080/10683160410001726356 (2005).

[29] Nahari, G. Verifiability approach: Applications in different judgmental settings. In *The Palgrave Handbook of Deceptive Communication*, 213–225. Springer International Publishing. http://dx.doi.org/10.1007/978-3-319-96334-1_11 (2019).

[30] Köhnken, G., Manzanero, A. L., & Scott, M. T. Análisis de la validez de las declaraciones: Mitos y limitaciones. *Anuario de Psicología Jurídica*, **25**, 13–19. https://doi.org/10.1016/j.apj.2015.01.004 (2015).
